# A Hypomethylated population of *Brassica rapa* for forward and reverse Epi-genetics

**DOI:** 10.1186/1471-2229-12-193

**Published:** 2012-10-20

**Authors:** Stephen Amoah, Smita Kurup, Carlos Marcelino Rodriguez Lopez, Sue J Welham, Stephen J Powers, Clare J Hopkins, Michael J Wilkinson, Graham J King

**Affiliations:** 1 Rothamsted Research, Harpenden, Herts AL5 2JQ, UK; 2 Current address: Southern Cross Plant Science, Southern Cross University, Lismore, NSW 2480, Australia; 3 Plant Research Centre, School of Agriculture, Food and Wine, Faculty of Sciences, University of Adelaide, Waite Campus, PMB1, Glen Osmond, SA, 5064, Australia; 4 Department of Pathology, The University of Melbourne, Melbourne, VIC, 3010, Australia

**Keywords:** DNA methylation, Hypomethylation, Epigenetics, Crop plants, Brassica, 5-Azacytidine

## Abstract

**Background:**

Epigenetic marks superimposed on the DNA sequence of eukaryote chromosomes provide agility and plasticity in terms of modulating gene expression, ontology, and response to the environment. Modulating the methylation status of cytosine can generate epialleles, which have been detected and characterised at specific loci in several plant systems, and have the potential to generate novel and relatively stable phenotypes. There have been no systematic attempts to explore and utilise epiallelic variation, and so extend the range of phenotypes available for selection in crop improvement. We developed an approach for generating novel epialleles by perturbation of the DNA methylation status. 5- Azacytidine (5-AzaC) provides selective targeting of ^5m^CG, which in plants is associated with exonic DNA. Targeted chemical intervention using 5-AzaC has advantages over transgenic or mutant modulation of methyltransferases, allowing stochastic generation of epialleles across the genome.

**Results:**

We demonstrate the potential of stochastic chemically-induced hypomethylation to generate novel and valuable variation for crop improvement. Systematic analysis of dose–response to 5-AzaC in *B. rapa* guided generation of a selfed stochastically hypomethylated population, used for forward screening of several agronomic traits. Dose–response was sigmoidal for several traits, similar to that observed for chemical mutagens such as EMS. We demonstrated transgenerational inheritance of some phenotypes. BraRoAZ is a unique hypomethylated population of 1000 E2 sib lines. When compared to untreated controls, 5-Aza C-treated lines exhibited reduced immuno-staining of ^5m^C on pachytene chromosomes, and Methylation Sensitive Amplified Polymorphism (MSAP) profiles that were both divergent and more variable. There was coincident phenotypic variation among these lines for a range of seed yield and composition traits, including increased seed protein content and decreased oil content, as well as decreased erucic acid and corresponding increases in linoleic and/or palmitic acid. Each 5-AzaC-treated line represents a unique combination of hypomethylated epialleles.

**Conclusions:**

The approach and populations developed are available for forward and reverse screening of epiallelic variation and subsequent functional and inheritance studies. The generation of stochastically hypomethylated populations has utility in epiallele discovery for a wide range of crop plants, and has considerable potential as an intervention strategy for crop improvement.

## Background

Epigenetic marks superimposed on the DNA sequence of eukaryote chromosomes have the potential to provide agility and plasticity in terms of modulating gene expression, ontology, and response to the environment. These marks affect chromatin structure, and include cytosine methylation of DNA and modifications to histone proteins. Epialleles have been detected and characterised at specific loci in several plant systems, resulting from variation at specific sites or in global patterns of DNA methylation or as histone variants [1]. Some *de novo* epialleles can confer novel heritable phenotypes over several generations [2,3]. An increasing body of evidence indicates that many crop agronomic traits are likely to be affected to some extent by stably inherited epigenetic modifications [3-5].

The extent to which inadvertent selection of epiallelic variation has contributed to the major increases in crop yield or quality achieved through selective breeding over the past century remains a mystery. Although epialleles have the potential to generate novel and relatively stable phenotypes [2,3,6], to date there have been no systematic attempts to explore and utilise epiallelic variation, and so extend the range of phenotypes available for selection in crop improvement. The scope for harnessing epigenetic variation is demonstrated by recent findings of an association between DNA methylation and selection for energy use efficiency in *Brassica* oilseed crops [4], as well as the interaction between phosphate starvation response in Arabidopsis and the pattern of histone H2A.Z epigenetic marks [5]. H2A.Z is excluded from sites of heavy DNA methylation in actively transcribed genes [7]. Tissue-specific monoallelic *de novo* DNA methylation at the *SP11* gene within the *Brassica rapa* pollen self-incompatibility locus *S* has been shown to contribute to dominance relationships amongst S-alleles [8]. In Arabidopsis, imprinted states mediated by changes in DNA methylation and histone chemistry affect vernalisation and flowering time [9,10], embryo development [11,12] and seed size [11].

DNA methylation is regulated by a set of genes that directly catalyse the methylation process or indirectly influence methylation status *via* chromatin remodelling. In mammals and other metazoans this is primarily achieved through the activity of *DNA METHYLTRANSFERASE 1* (*DNMT1*) which encodes the enzyme responsible for ^5m^C maintenance, and *DOMAINS REARRANGE METHYLASE 1* (*DRM1*) which encodes enzymes responsible for *de novo* methylation at CG sites. Plants differ in the targets, distribution and transmission of DNA methylation compared with animals. The Arabidopsis *DNA METHYLTRANFERASE 1* (*MET1*) is required for catalysing cytosine methylation in CG context, resulting in ^5m^CG [13,14], whereas the chromatin remodelling factor *DECREASE IN DNA METHYLATION 1* (*DDM1*) is required for maintaining DNA methylation both at CG and CHG sites [15].

Specific patterns of DNA methylation can be transmitted through mitosis and cell lineages, as well as transgenerationally through meiosis, mediated by *MET1*[16,17]. The stability of epiallelic variation has been demonstrated in a number of plant systems, including *Brassica napus*[18]. In Arabidopsis, epiRILs have been established with variation and high heritability for flowering time and plant height, as well as stable inheritance of multiple parental DNA methylation variants over at least eight generations [19]. Subsequent phenotyping has demonstrated heritable variation for many fitness traits [20]. Whole genome analysis of DNA methylation in Arabidopsis has shown that CG sites tend to be targeted within coding sequences [21,22]. More recently, it has been found that these marks appear preferentially associated with exons [23].

One approach to generating novel epialleles is by perturbation of the DNA methylation status [24,25]. This may include ectopic, tissue-specific or induced silencing of DNA methyltransferases and other methylation regulators in the genome. In Arabidopsis, down-regulation of *MET1* results in a wide range of abnormal phenotypes, including decreased plant stature, smaller rounded leaves, decreased fertility and reduced apical dominance [6,14]. Although this has provided valuable insights into the role of cytosine methylation (^5m^C) in gene function and development, there are drawbacks to employing *met1* mutants in the context of crop improvement, due to the widespread re-patterning of epigenetic marks within the genome. However, as with conventional functional gene analysis, there is potential to reveal a range of variants that may underpin epigenetic modulation of specific genes or traits. Such an approach would be facilitated by exploring a range of epialleles associated with specific target genes (reverse epigenetics) or phenotypic traits (forward epigenetics).

Targeted chemical intervention, using demethylating agents such as 5-Azacytidine (5-AzaC), provides an alternative to transgenic or mutant modulation of *MET1*. 5-AzaC is a structural analogue of the nucleoside cytidine with nitrogen atoms in place of carbon in the fifth position of the ring structure [26,27]. It is a potent inhibitor of DNA methyltransferases and has been shown to be effective in reverting the hypermethylation of tumour suppressor genes and suppressing cancer-specific cellular phenotypes [26,28-31]. Since 5-AzaC interferes with MET1, there is a selective targeting of ^5m^CG, which in plants is associated with exonic DNA. The use of 5-AzaC has a number of advantages over direct down-regulation of *MET1*, including potential for stochastic hypomethylation of target sites, rather than widespread hypomethylation that may mask more subtle and valuable phenotypes, or result in lethal epialleles. This presents the opportunity to screen for specific epialleles that may then be stably introgressed into a wild type background. Naturally occurring epialleles have recently been shown to occur in near-isogenic lines of maize and to exhibit relatively stable inheritance [32].

Epigenetic intervention through generation of novel epialleles may be particularly tractable in the many crop plants of polyploid origin, where there is additional scope for paralogue- or homoeologue-specific gene regulation. The ability to sustain non-lethal variation is evident from the higher mutant loads attainable in EMS TILLING populations for *B. rapa*[33], *B. napus*[34] and wheat [35]. Species within the genus *Brassica* have been domesticated into a remarkably wide range of vegetable, oilseed, fodder and condiment crops [36], including turnips, Chinese cabbage, pak choi, brocolleto, sarson and turnip rape of *B. rapa* (A genome, n=10) and cabbage, cauliflower, broccoli, Brussels sprout of *B. oleracea* (C genome, n=9). The A genome is retained essentially intact within the amphidiploids of *B. napus* (AC genomes, n=19) giving rise to the major crops oilseed rape, Canola and swede/rutabaga and mustard rape/brown mustard of *B. juncea* (AB genomes, n=18). It remains unclear why *Brassica* species have the capacity to generate such a wide range of morphological forms, although this has been attributed to gene duplication, intergenomic heterozygosity and epigenetic phenomenon [37-39]. In the absence of direct evidence from whole genome bisulphite sequencing, estimates of ^5m^C methylation within *Brassica* have been based on immunoprecipitation, giving estimates of 16% of all cytosines within the genome [40], and methylation sensitive amplified polymorphism (MSAP) giving estimates from 16-57% of target sites [40-42].

Here, we demonstrate the potential of stochastic chemically induced hypomethylation to generate novel and valuable variation for crop improvement. We systematically optimise the phenotypic dose–response to 5-AzaC in *B. rapa* and demonstrate the transgenerational inheritance of some phenotypes. We then use this information to generate a stochastically hypomethylated population which was selfed and characterised for a number of agronomic traits including seed size and composition. We demonstrate changes in the distribution of ^5m^C in euchromatin of pachytene stage chromosomes, variation in MSAP profiles and alterations in the transcriptome. The approach and populations developed are available for forward and reverse screening of epiallelic variation and subsequent functional and inheritance studies.

## Results

### Plants phenotypes have a sigmoidal dose–response to 5-AzaC hypomethylation

Dosage response of the plants was investigated after exposure to various concentrations of 5-AzaC (water control, 0.01mM, 0.10mM, 0.50mM, 1.00mM and 1.50mM) during seed imbibition by examination of a range of phenotypic traits during plant development.

**Figure 1:**
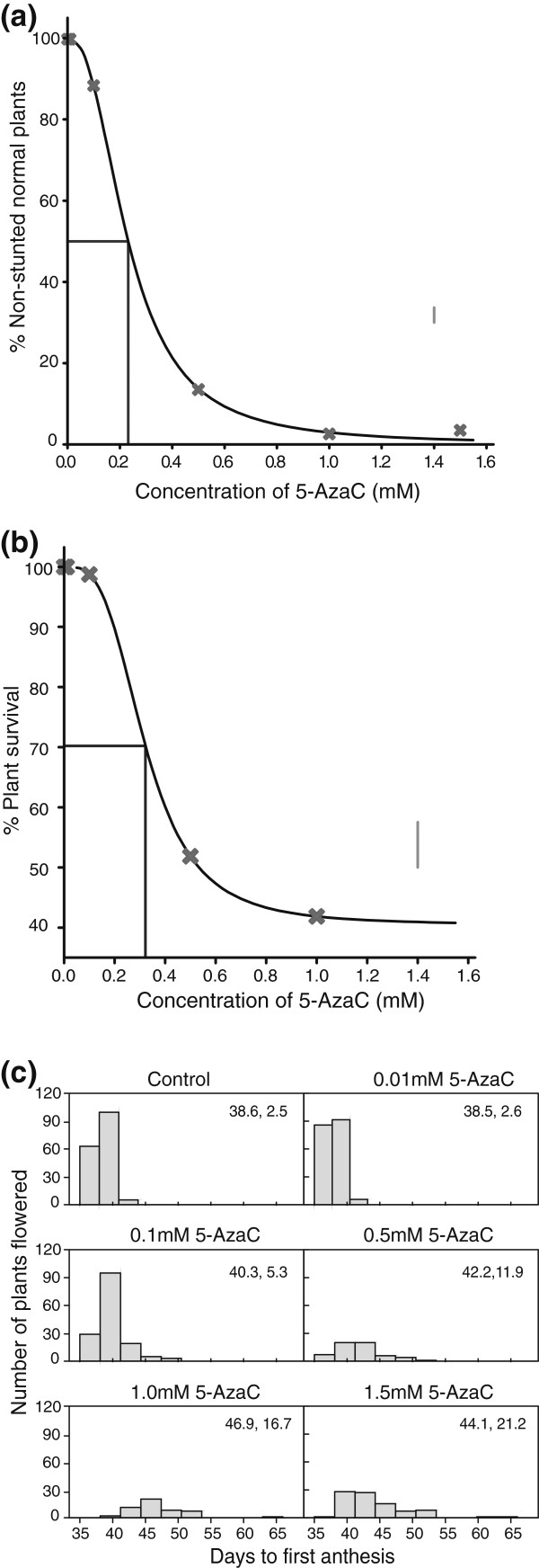
**Phenotypic response of *B. rapa *R-o-18 to 5-AzaC treatment at E1 generation.** Lines drawn to the curve indicate the dose giving 50% reduction in normal plants. The vertical line at 1.4mM indicates LSD (5%) values for comparison of means. The plotted points in (**a**) and (**b**) show the means of four replicate observations at each concentration. (**a**) Dosage response curve of plant stature 20 days after sowing. Lines drawn to the curve indicate the concentration that reduces survival by 50% of the overall possible reduction given an estimated (significant, p ≤ 0.05) lower asymptote. The vertical line at 1.4mM indicates LSD (5%) values for comparison of means. (**b**) Survival curve 60 days after sowing. (**c**) Frequency distribution of days to first anthesis. Mean and variance are indicated in top right of each panel.

Germination of E1 seed was not significantly affected (p>0.05) after being imbibed in 5-AzaC at concentrations up to 1.0mM, although there was a small but significant decline (to 98% germination) following exposure to 1.5mM (p ≤ 0.05). During the early stages of development, there were clear differences in gross phenotype in terms of stature and rate of growth (Figure 1). Plant growth was markedly stunted at concentrations of 5-AzaC above 0.1mM (Additional file 1: Figure S1 and Figure 1a). In some seedlings that failed to develop true leaves, root development was poor and plant stature conspicuously reduced.

There was a significant effect on days to first anthesis (Figure 1c and Additional file 1: Figure S2), at 0.1 mM and above (p < 0.05). In general, there was an increase in days to flowering with increasing concentrations of 5-AzaC. The variance in days to flowering also increased with 5-AzaC concentration. Reduced pollen productivity and increased incidence of defective anthers was observed at higher 5-AzaC concentrations, although all plants with seemingly viable pollen were able to set at least some seeds. However, there was an overall reduction in seed set and seed weight associated with increased 5-AzaC concentration (Table 1 and Additional file 1: Figure S3).

**Table 1:** Effect of 5-AzaC treatment on seed set and seed weight

	**Silique 6**				**Silique 7**				**Silique 8**			
	**Seed set**		**Seed weight (mg)**		**Seed set**		**Seed weight (mg)**		**Seed set**		**Seed weight (mg)**	
	**Mean (s.e.)**	**Var.**	**Mean (s.e.)**	**Var.**	**Mean (s.e.)**	**Var.**	**Mean (s.e.)**	**Var.**	**Mean (s.e.)**	**Var.**	**Mean (s,e.)**	**Var.**
Control	31.05 (2.35)	110.16	4.37 (0.17)	0.56	35.11 (2.16)	88.65	4.38 (0.16)	0.48	35.20 (1.91)	72.80	4.41 (0.11)	0.24
0.01mM	31.70 (2.50)	125.38	4.40 (0.14)	0.37	32.65 (2.21)	97.82	4.37 (0.11)	0.26	32.45 (2.65)	140.37	4.37 (0.12)	0.29
0.1mM	19.80 (2.60)	135.64	4.38 (0.24)	1.11	24.25 (2.51)	125.67	4.39 (0.17)	0.60	21.55 (2.87)	165.21	4.48 (0.19)	0.74
0.5mM	23.40 (3.19)	203.83	4.04 (0.21)	0.87	22.65 (2.83)	159.92	4.01 (0.23)	1.01	24.25 (3.09)	191.36	4.12 (0.18)	0.67
1.0mM	15.25 (2.62)	137.36	2.61 (0.39)	2.97	15.40 (2.48)	122.88	2.61 (0.38)	2.85	19.90 (2.50)	125.15)	2.87 (0.38)	2.85
1.5mM	22.65 (2.51)	125.82	3.86 (0.23)	1.04	19.40 (3.17)	201.09	3.61 (0.31)	1.94	21.05 (2.52)	127.31	3.99 (0.27)	1.45
SED	4.73		0.29		4.03		0.28		4.31		0.36	
df	15		12		15		13		15		14	

Response of *B. rapa* seed set (number of seeds per silique) and seed weight (mg) to 5-AzaC treatment. Plants were grown from seeds treated with different concentrations of 5-AzaC (0.01mM, 0.1mM, 0.5mM, 1.0mM and 1.5mM) and a control. Seeds were harvested from nominated positions (sixth, seventh and eighth positions, from the apex of the primary inflorescence) of randomly selected E1 plants. A total of 20 plants in each concentration, consisting of four replications (five plants per replicate) were used for this analysis. The data were analysed by ANOVA and the table shows the mean, standard error of the mean, variance of observations, the standard error of the difference (SED) between means and the degrees of freedom (df).

The effect of 5-AzaC exposure also carried forward into the seminal generation. There was a small but significant (p ≤ 0.05) reduction in E2 seed germination; down to 92% at concentrations of 1.0 mM 5-AzaC and above. Compared to plants from control seeds where anthesis occurred at 35 days, there was also a reduction of 2.8 days (p ≤ 0.01) to first anthesis in a subset of E2 plants at 0.5 mM 5-AzaC. Some E2 plants also exhibited chlorophyll sectoring.

Careful analysis of two dose–response curves (Figure 1a and 1b, relating to abnormal phenotype and survivorship) allowed selection of optimal concentrations of 5-AzaC to generate a hypomethylated population in *B. rapa* (Figure 1a, b). There was no significant (p > 0.05) lack of fit of the data for either of these curves. This test was based on a total of 24 observations for data in Figure 1a (four replicate observations at each of six concentrations), and 20 observations for data in Figure 1b (four replicates at five concentrations). A concentration of 0.23 mM represented the concentration at which no more than 50% of the plants exhibited a phenotype of reduced stature and/or growth rate (Figure 1a). At a concentration of 0.32 mM 5-AzaC around 70% of the population are expected to survive (Figure 1b); this being the concentration that reduces survival by 50% of the overall possible reduction given an estimated (significant, p ≤ 0.05) lower asymptote. Accordingly, we adopted two concentrations, 0.25 mM and 0.30mM 5-AzaC for generation of the BraRoAZ population.

### Establishment of the BraRoAZ DNA hypomethylated population (E1 to E3)

**Figure 2:**
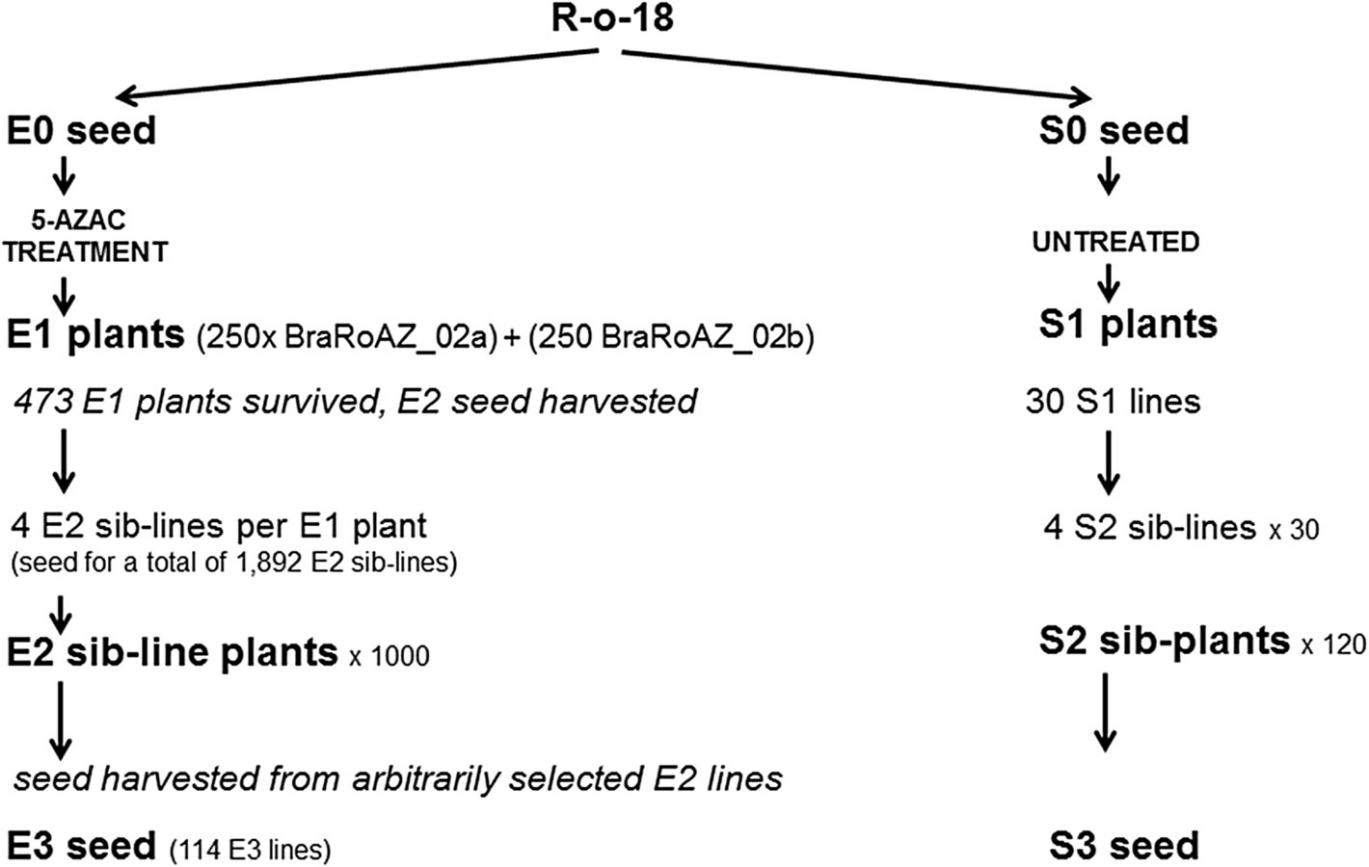
**An overview of the 5-AzaC-treated population BraRoAZ.** A total of 500 E1 plants was raised from (E0) seeds treated with 0.25mM or 0.30mM 5-AzaC. From each of 473 E1 plants, seed were harvested from three siliques and assigned independent accessions. Seed were harvested from the remaining siliques and combined. 10 seeds (sib-lines) were sown from each of a subset of 100 E1 plants, selected based on morphological features such as plant stature, branching and floral morphology. From these E2 plants, 114 were randomly selected to obtain E3 seed. Single siliques were harvested from each plant, and the remaining seed were combined.

Having identified suitable concentrations of 5-AzaC that would generate a population comprising plants that are likely to be fertile and yield good seed, and yet also show heritable phenotypic effects, we then applied these concentrations to establish a hypomethylated E1 population of *B. rapa* line R-o-18 (BraRoAZ). Two sub-populations, each containing 250 plants, were established (BraRoAZ_02a and BraRoAZ_02b) from seed treatments of 0.30mM and 0.25mM 5-AzaC respectively (Figure 2).

**Figure 3:**
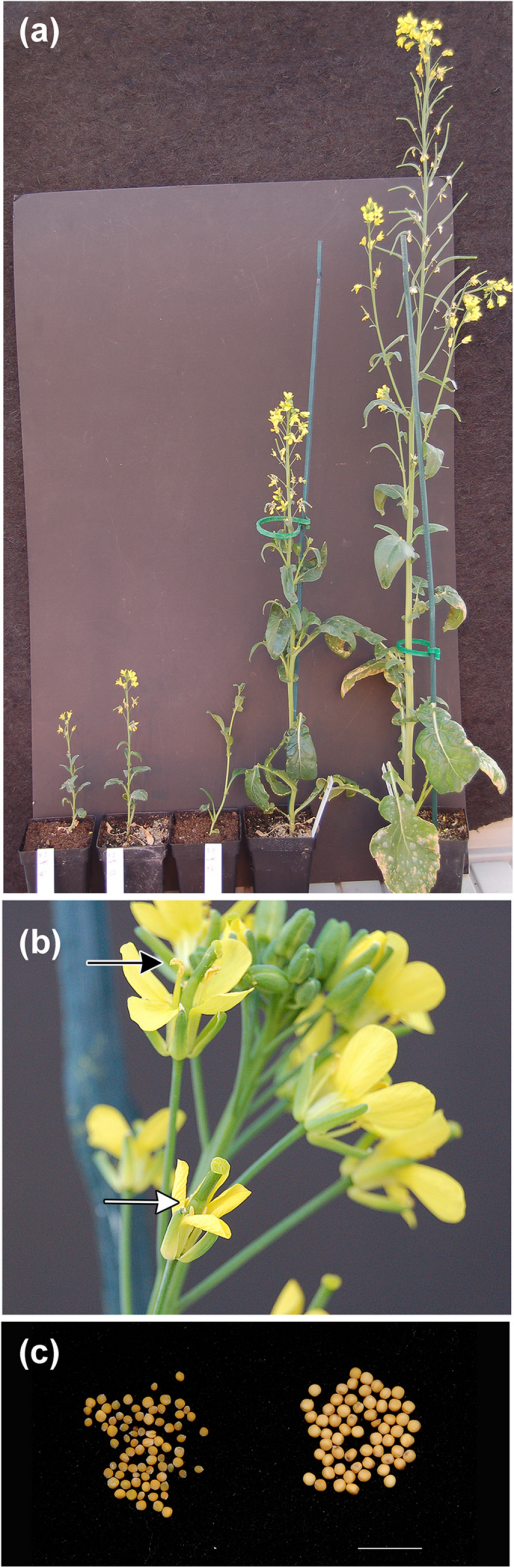
**Morphological variation observed in E1 plants from treatment with 5-AzaC at 0.25mM and 0.3mM.** (**a**) Plants showing a continuum of plant stature and an example of a late flowering phenotype. (**b**) normal anthers (black arrow) and male sterile anthers (white arrow) were frequently observed on the same plant. (**c**) Reduced seed size in E3 sib line BraRoAZ_11714e3 (left panel) compared to R-o-18 (right panel). Bar= 1cm.

Seed for a total of 1,892 E2 sib-lines were harvested. Phenotypic comparison of the E1 plants with the control (untreated) plants (Figure 3) allowed selection of 1,000 E2 sib-lines (500 per original treatment) (Figure 2) for assessment of the E2 generation. These corresponded to 50 original E1 plants per treatment, where atypical variation (i.e. beyond that seen in the WT controls) had been observed in survival rate, flowering time, plant height, secondary branching and floral morphology.

E2 selfed seed were harvested from 473 E1 lines, together with 30 control (untreated parental) lines. For each E1 plant, seed from three separate siliques from the middle of the primary inflorescence were harvested and stored independently. In addition, seed from the remaining siliques for each plant were pooled. One seed was used per seed source to generate the E3 generation. Thus, there were four unique E2 seed accessions (E2 sib-lines) per E1 plant (Figure 2).

In general there was a lower level of morphological phenotypic variation in the E2 plants compared with the E1 plants but still higher than the controls. However, we did observe a low frequency of sectoring, reduced plant stature, smaller leaves, defective anthers, embryo abortion, reduced seed set and seed size.

We next harvested selfed seed from 114 arbitrarily selected E2 sib-lines (from the 1,000 grown) corresponding to 28 original E1 plants, to generate an E3 population. Seed from one selected silique were harvested and stored independently, with the remaining seed pooled. These plants retained a higher variability than was seen in the controls, albeit less than the E1.

### Variability in patterns of DNA methylation

**Figure 4:**
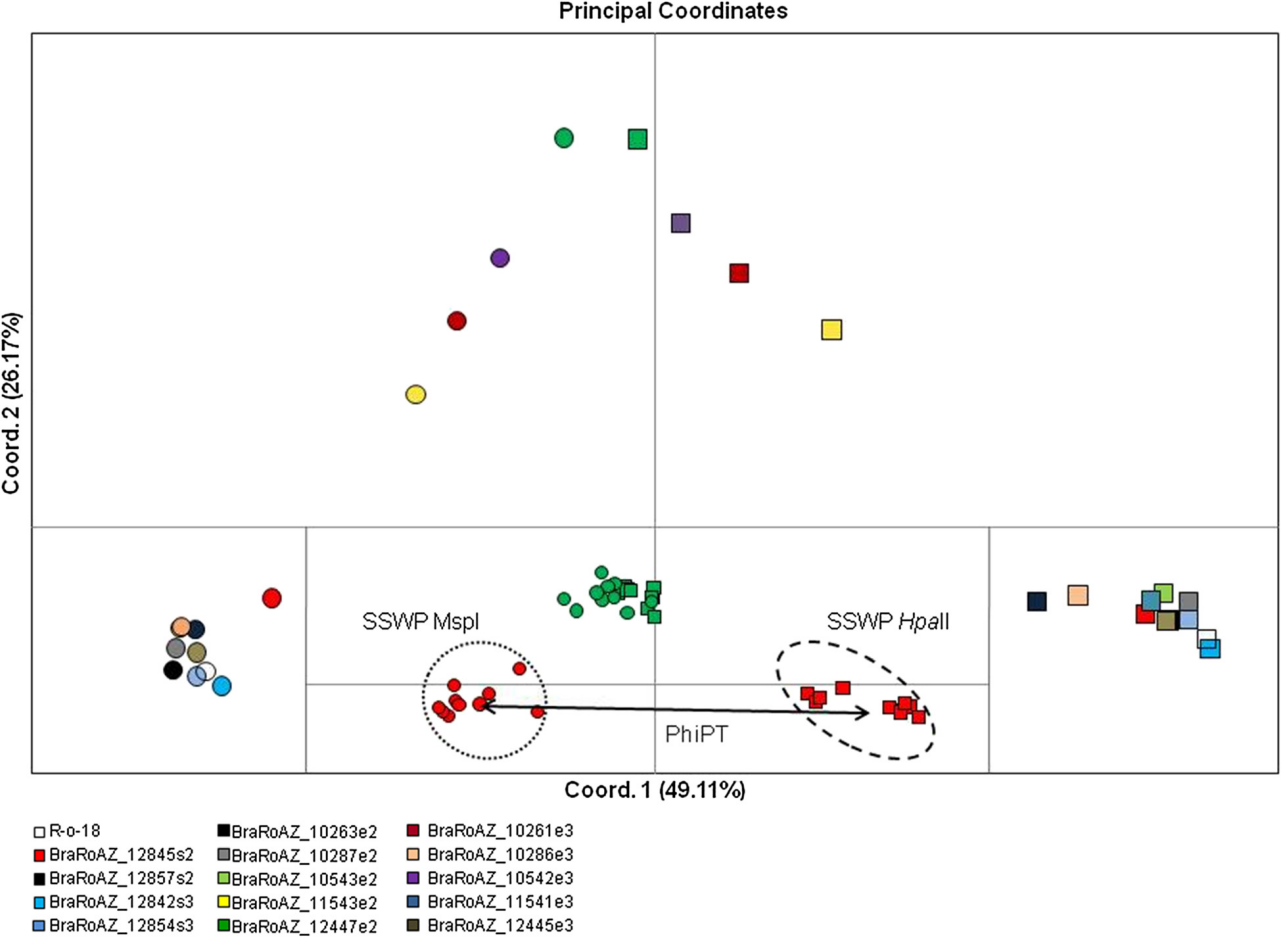
**Effect of 5-Azac on epigenetic instability.** Principal coordinate analysis (PCoA) based on the Euclidean analysis of methylation-sensitive amplified polymorphism (MSAP) assays using primer combinations H2/E1 and H3/E3. Plant lines are colour coded as shown in key, with square symbols (*Hpa*II) and circles (*Msp*I). **a**) Main graphic, PCoA showing mean epigenetic distances for each of the groups; **b**) Nested graphic, epigenetic distances for all the samples in lines BraRoAZ_12447e2 and BraRoAZ_12845s2. Euclidian space within circles represent intra-population variation (i.e. SSWP) for control line BraRoAZ_12845s2 using *Hpa*II (dashed) and *Msp*I (dotted). Arrow indicates the epigenetic distance between BraRoAZ_12845e2 samples restricted with *Hpa*II or *Msp*I (PhiPT).

MSAP analysis was used to assess the extent to which treatment had perturbed global methylation profiles. The analysis was based on a sample of 10 seeds from five lines each of S2, E2 and E3 and two S3 lines. The capacity of 5-AzaC to inhibit methyltransferase activity led to the expectation that profiles generated by the methylation-sensitive *Hpa*II and *Msp*I would diverge in untreated samples (since methylation will disproportionally affect the former profiles) but converge in treated samples (since the DNA templates lack the methylation that cause the difference). As expected, there was marked divergence revealed by Principal Coordinate Analysis (PCoA) between the *Hpa*II and *Msp*I profiles in all untreated samples (Figure 4). However, there was considerable variance between the behaviour of the treated lines. Four treated lines (BraRoAZ_11543e2, BraRoAZ_12447e2, BraRoAZ_10261e3, BraRoAZ_10542e3) from the E2 and E3 generations showed a marked loss in discrimination between enzyme profiles relative to the untreated controls (Figure 4). Conversely, the remaining treated lines retained the wide divergence between the *Hpa*II and *Msp*I profiles, and so were indistinguishable from the untreated control lines (Figure 4). The significance of these differences was estimated using PhiPT by AMOVA. Here, the same four lines were alone in generating sufficiently low PhiPT values to infer non-significant divergence between the *Hpa*II and *Msp*I profiles (0.1642<P>0.0006). All remaining lines produced the *Hpa*II and *Msp*I profiles that were highly significantly divergent (P=0.0001) (Additional file 2: Table S2).

**Figure 5:**
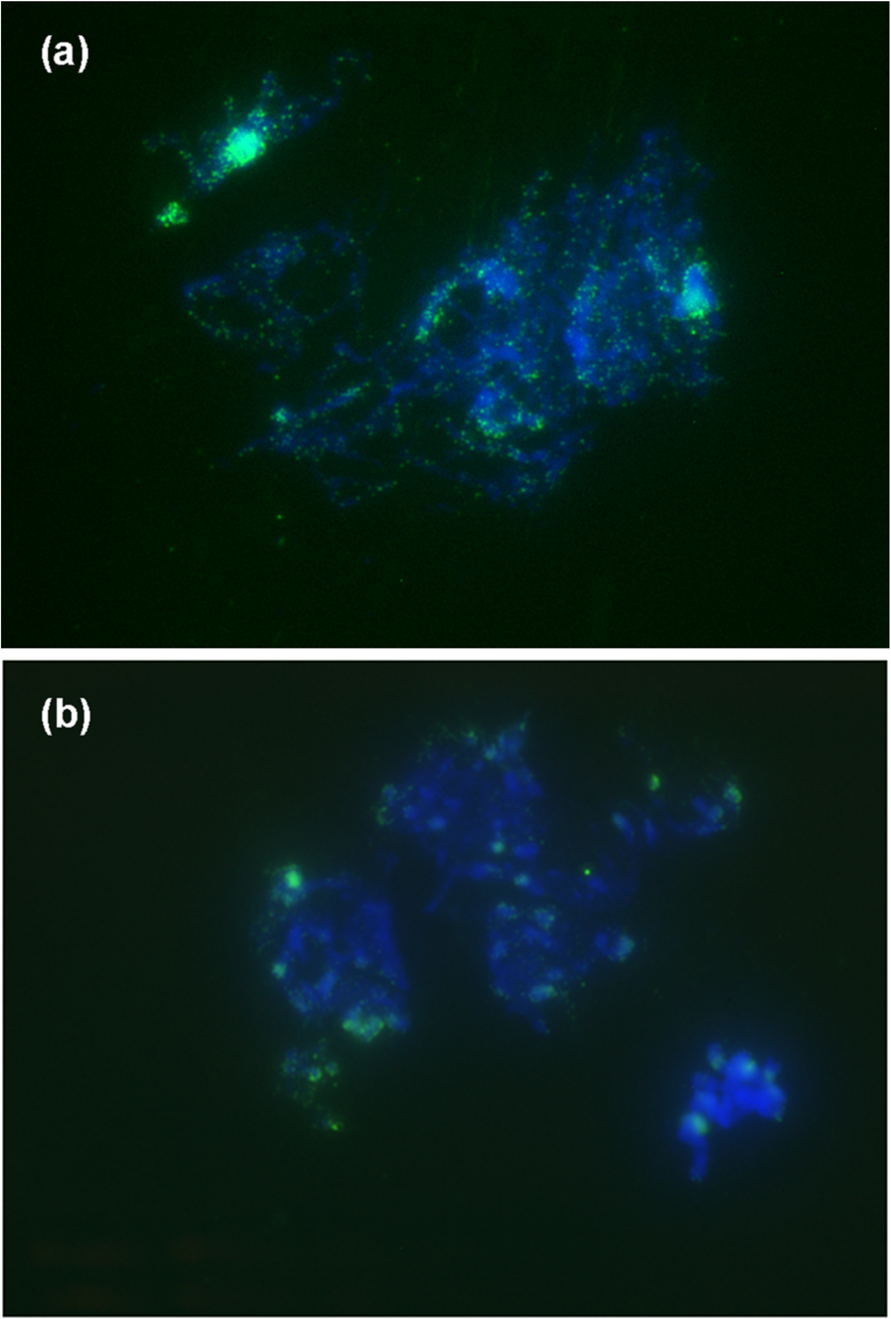
**Immunodetection of**^**5m**^**C in (a) wild type S2 and (b) E2 pachytene chromosome spreads from meiocytes.** 4’,6-Diamidino-2-Phenylindole (DAPI) staining indicated in blue, and anti-5mC detected using Alexa-488 conjugated secondary antibody indicated in green. E2 (**b**) shows reduced ^5m^C signal in non-heterochromatic regions compared with S2 (**a**).

We assessed the spatial distribution of ^5m^C in chromosomes by immuno-labelling in meiotic pachytene spreads of control S2 lines, and in four BraRoAZ_E2 lines. Clusters of ^5m^C signal co-localised with heterochromatic regions both in control plants and in BraRoAZ_E2 plants but were notably reduced within the euchromatin of the BraRoAZ_E2 lines (Figure 5).

### Hypomethylation affects seed size and other yield components

**Figure 6:**
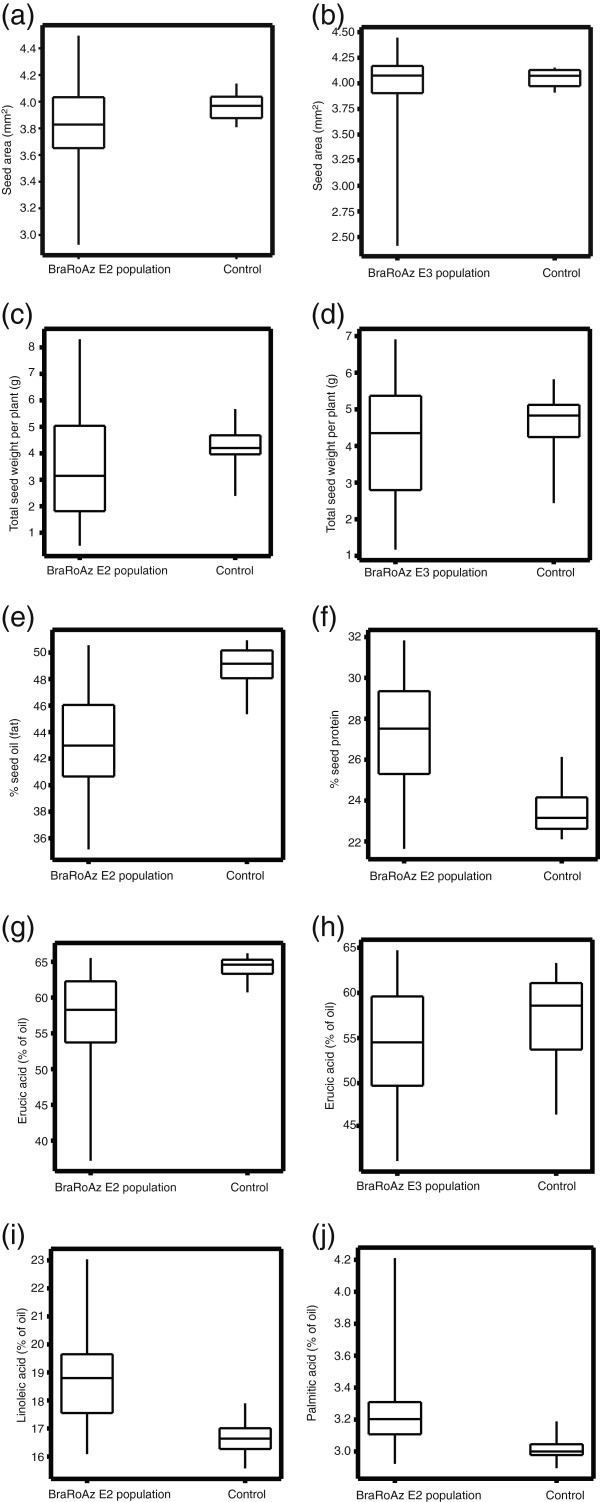
**Boxplots displaying difference in seed size and composition traits recorded in the BraRoAZ E2 and E3 populations compared to control S2 or S3 populations.a**) and **b**) Seed area recorded in the E2 population compared to S2 control and E3 population compared to S3 controls, respectively. **c**) and **d**) show total seed weight per plant for the E2 and E3 populations. **e**) total oil content as a percentage of dry weight recorded in E2 compared to S2. **f**) total protein as a percentage of dry weight recorded in E2 compared to S2. **g**) and **h**) erucic acid as a % of the total seed oil measured by NIRS for E2 and E3 with respective controls. **i**) linoleic acid shows an increase in E2 compared to S2 and **j**) palmitic acid as a % of total oil content in E2 compared with S2 control.

We next examined whether agronomically important aspects of seed morphology and composition had been altered by the treatment. Seed size was inferred indirectly from the area of seed in profile. Seeds from BraRoAZ E2 lines were on average 0.14mm^2^ (SE 0.040) smaller than the S2 controls (Figure 6a; Wald statistic = 11.97 on 1 df; p <0.001). Components of variation between lines were greater for the BraRoAZ E2 lines (variance = 0.125) than for the control R-o-18 S2 sib-lines (variance = 0.009, change in deviance compared to common variance = 40.4 on 1 df, P<0.001). Similarly, variation between seeds within lines was greater for the BraRoAZ E2 lines (variance = 0.122) than for the control R-o-18 S2 sib-lines (variance = 0.083, change in deviance compared to common variance = 40.4 on 1 df, P<0.001), and a subset of BraRoAZ E2 lines exhibited a much greater range of seed size. BraRoAZ E3 lines also had a greater range of seed size compared with the S3 controls (Figure 5b). We found no evidence of correlation between seed size and seed number per silique in individual sib-lines and control lines.

Seed yield within the BraRoAZ_E2 and BraRoAZ_E3 populations was considerably more variable than in the corresponding S2 and S3 lines (Figure 6c, d). We analysed a subset of 100 E2 lines and found that seven of the top 10 yielding lines were scored with profuse branching (increase in number of axillary branches subtending main stem) compared with nine of the remaining 90 lines. Seed yield per plant showed a strong correlation (r^2^=0.94) with the number of seeds per plant, which may be a direct consequence of the increased branching and/or increased fertility. However, the observed variation in seed size was poorly correlated with seed yield (r=−0.12), as well as with number of seed per plant (r=−0.32). This suggests that the effects of treatment on seed size are probably independent from those on other yield components.

### Seed composition

The BraRoAZ E2 sib-lines yielded seed with a lower total oil content (Figure 6e; E2=43.1%, S2=48.9%, Wald statistic = 5261.3 on 1 df, P<0.001) and higher total protein content (Figure 6f; E2=27.2%, S2=23.4%, Wald statistic = 4475.4 on 1 df, P<0.001) than the S2 controls. For both components there was a similar range of variation within the BraRoAZ_E3 population.

There was increased variation in proportion of individual fatty acids in the BraRoAZ E2 population compared with the controls (Figure 6g,l,j). Within the BraRoAZ E3 population we observed a similar, but more extenuated variability, although here, increased spread was also observed in the S3 control lines, perhaps indicating greater GxE interactions (Figure 6h). In general, there was an increase in palmitic (E3=3.24%, S3=3.15%, Wald statistic = 60.2 on 1 df, P<0.001) and linoleic (E3=19.45%, S3=18.82%, Wald statistic = 88.2 on 1 df, P<0.001) acids, corresponding to a decrease in erucic acid (E3=54.19%, S3=57.56%, Wald statistic = 629.9 on 1 df, P<0.001) in most treated lines. Examination of the pairwise relationships between line means for individual fatty acids enabled us to infer that hypomethylation may have targeted specific steps in fatty acid elongation and/or side-chain modification [43]. For example, the negative correlation (r= −0.88) of erucic acid : linoleic acid is consistent [43] with up-regulation of *FAD2*. Furthermore, there was a positive correlation (r=0.75) between palmitic acid and linoleic acid, and a negative correlation (r=−0.74) between palmitic acid and erucic acid.

### Stochastic hypomethylation and associated transcriptional changes in *B. rapa*

Finally, we sought evidence for specific transcriptional changes associated with 5-AzaC-induced hypomethylation in a representative treated *B. rapa* line. In these experiments, leaf RNA from two wild-type control lines and a single E3 line (BraRoAZ_12445e3, selected based on a phenotype of reduced seed size compared with S3 seed) was hybridised against the Affymetrix GeneChip® *Brassica* Exon 1.0 ST Array, which is based on the 135k unigene set [44]. At a threshold of 1.2- fold change in transcription level, 10% of genes appeared to show a change in transcript level. At a higher threshold of 2-fold change, 3.7% of genes showed a change in transcript level, with 216 genes up-regulated in the BraRoAZ_12445e3 plant compared with the R-o-18 wild-type control (Additional file 3: Table S3). Preliminary analysis of GO terms indicated that 79 genes classified as involved in the cellular component were upregulated in the BraRoAZ plant. Overall, a small number of genes were significantly up-regulated in this BraRoAZ plant (two fold threshold), while none appeared to be significantly down-regulated.

## Discussion

We have performed a systematic dose–response characterisation of the effect of DNA hypomethylation induced by 5-AzaC in *B. rapa*. Based on this analysis, we generated a unique hypomethylated population of 1,000 E2 sib-lines with a stochastic distribution of reduced ^5m^CG marks. Each line represents a unique combination of hypomethylated epialleles that are available for forward and reverse genetic screening. We carried out forward screening and investigated the range of variation for seed size and other yield components, as well as seed composition. These epi-TILLING populations are analogous to those generated using conventional nucleotide mutagens such as EMS [33,45]. However, they do offer the distinct opportunity to explore and exploit subtle interactions with phenotype that may be associated with epiallelic variation.

Before adopting any treatment that seeks to reveal new epigenetic variation through interference in endogenous methylation control systems, it is important first to demonstrate efficacy of treatment and to optimise its application. 5-AzaC is a structural analogue of the nucleoside cytidine [26,27] that inhibits the action of the DNA methyltransferase MET1, which in plants maintains methylation in exonic ^5m^CG contexts. However, there is scope for ineffective application should the 5-AzaC fail to access the target tissues, or should the time of exposure be insufficient to elicit a response. Conversely, excessive application could lead to toxicity and/or lethal levels of mutation or demethylation [46]. It is therefore important to develop appropriate strategies to evaluate whether exposure levels were sufficient to ensure efficacy of demethylation whilst also minimising potentially damaging effects. We used frequency of survivorship and the absence of gross phenotypic abnormality to establish that the level of 5-AzaC applied was not excessive. The dose–response to 5-AzaC was sigmoidal for several traits recorded, similar to that observed for chemical mutagens such as EMS. This suggests that the *B. rapa* genome is able to tolerate low concentrations up to ~0.1mM 5-AzaC for the treatment period of three days during which mitoses are occurring in the meristematic cells. Above this concentration, an exponential response is observed up to ~1.0mM 5-AzaC, above which there were no apparent additional lethal effects. Moreover, there were only minor cytotoxic effects observed within hypocotyls and cotyledon tissues in seedlings. In general, the E1 plants completed their life cycle at all concentrations, and successfully set seed.

The potency of the 5-AzaC dose–response is likely to vary, depending on the dinucleotide composition and structural organisation of different genomes [29]. Compared with EMS in the same R-o-18 genotype [33], 5-AzaC gave rise to a greater proportion of plants with a normal visual phenotype, although the effects on fertility were similar.

It is important next to verify that the 5-AzaC treatment apparently leading to these effects is doing so by causing hypomethylation of the genome. We examined this in two ways. First, immune-localisation of methylated cytosines revealed essentially similar distributions of FISH signal across the euchromatic regions of pachytene chromosomes in treated (E2) and control plants but with a marked reduction of signal in treated lines. This is consistent with the expected genome-wide hypomethylation associated with 5-AzaC treatment and congruent with previous observations in Triticale [47]. Second, we compared the level of disparity between the MSAP product profiles generated by the isoschizomers *Hpa*II (methylation sensitive) and *Msp*I. As expected, untreated control lines exhibited wide divergence between the profiles generated by the two enzymes, largely because of their differential sensitivity to the presence of cytosine methylation. However, it was notable that this distinction had been lost in four of the treated lines sampled, but had been retained in others. The most plausible explanation of this finding is that hypomethylation triggered by the 5-AzaC treatment had been stochastically effective and was only apparent in a subset of treated plants. This finding is consistent with the similar frequency of phenotypic abnormality seen in the E2 and E3 plants. Given the stochastic nature of 5-AzaC efficacy, this relatively simple approach has direct and valuable utility in allowing early identification of E1 lines where the treatment has actually affected the intended hypomethylation. This capacity would greatly improve the efficiency of any high-throughput programme seeking to use the chemical to generate new levels of phenotypic variation, or otherwise to manipulate the methylation status for genetic or epigenetic studies of gene function.

Having generated variation in methylation status, it is important next to determine the extent of associated phenotypic variation among the treated plants. In the present study, we certainly found additional phenotypic variation at the E1 generation when compared to untreated controls, as reported previously in a range of plant species [10,48,49]. However, compared with *B. oleracea*[50], 5-AzaC appeared to give rise to a reduced range of morphological phenotypic effects in *B. rapa* R-o-18. This may be partly attributed to the smaller genome size of the *Brassica* A genome [51] and/or different chromatin organisation, particularly in terms of relative amounts of heterochromatin and/or distribution of transposable elements [52]. Using a cytological approach, Braszewska-Zalewska [53] showed that the *B. rapa* genome has a distinct distribution of DNA methylation, primarily located in the heterochromatin when compared with *B. oleracea*.

There was also evidence that novel variation was present in several traits of agronomic importance. For example, flowering time is a complex multigenic trait that is regulated through the interaction of different signal transduction pathways [54-56]. Our finding that flowering time in the E1 generation is delayed following exposure to ≥ 0.1mM 5-AzaC is consistent with methylation playing some role in the control of flowering time in *B. rapa*. The clone of *B. rapa* used (R-o-18) is unresponsive to vernalisation, in common with the Arabidopsis ecotype Landsberg *erecta*[57]. This phenotype in Arabidopsis has been attributed to reactivation of FWA, a repressor of flowering which is constitutively methylated in its promoter and therefore repressed in the wild type at most stages of the life cycle. FWA is maternally imprinted [58] and is dependent on DNA methylation for its imprinted state [59,60]. However, we also observed a reduction in the time to flowering in a subset of E2 plants exposed to 0.5 mM 5-AzaC, a finding that is congruent with several previous reports [10,48,50,61] Subsequent systematic screening of flowering time within the BraRoAZ E2 population treated with 0.25 mM 5-AzaC also indicated a reduced time to flowering. Curiously, in some cases early flowering phenotypes were observed in the E2 generation from E1 lines had been late flowering, a phenomenon that has also been found in E2 and E3 populations of *B. oleracea* var. *italica* (King, unpublished data). This may be due to segregation of alleles in the E2 generation that affect different components of the flowering pathway and is perhaps indicative of the high level of redundancy and interdependency in the epigenetic control of this important trait.

The increased variability generated by stochastic hypomethylation of the genome has most value if this variation can be fixed or at least stabilized. Previous studies have demonstrated that this can be achieved in Arabidopsis through self-pollination [19] or doubled haploid production (King, unpublished). The dose–response analysis informed our selection of optimal concentration of 5-AzaC to use for generation of the BraRoAZ population, where E2 seed was set from 94.5% of E1 plants. The establishment of this population is analogous to an EMS-TILLING population and is available to be used for both forward and reverse epi-genetic screening. As expected, we observed a reduced range of phenotypes compared with EMS mutagenesis in the same R-o-18 genotype [33]. 5-AzaC only targets a subset of methylated sites where these exist, whereas EMS induces transition mutations which can lead to truncation of transcripts and modification of proteins, and is able to target all regions of the genome.

In plants, DNA methylation is at least sometimes transmitted directly through meiosis and maintained through post-meiotic mitosis giving rise to gametophytes [62,63]. In two separate experiments (dose–response and BraRoAZ population) we found that certain phenotypes were transgenerationally inherited from E1 to E2 generation following 5-AzaC treatment. These included an overall reduction in plant stature, small seed size and modified floral morphology. The apparent loss of variation observed in E2 and E3 generations relative to E1 could arise for a number of reasons, including re-methylation of specific cell-lineages forming gametes, dominance of wild-type alleles and segregation of epistatic loci. The E2 and subsequent generations are expected to possess functional *MET1* loci, and hence progressive re-methylation of specific CG sites may occur. The ability to distinguish between novel epi-alleles and collateral genetic variation, such as may result from activation of transposons, requires more detailed segregation and molecular analyses.

The range of morphological variation we observed was comparable to that observed following down-regulation of genes involved in maintenance DNA methylation [6,14,64]. A key attribute of the hypomethylated population we have generated is that there has only been a transitory exposure to 5-AzaC, that inhibits MET1 activity for the small number of cell divisions that occurred over three days during seed imbibition and meristem activation. In contrast, constitutive down regulation of *MET1* (in mutants or by RNAi) affects a range of phenotypes including plant stature, leaves, apical dominance, flowering and fertility [6,14,65]. From preliminary analysis of EMS mutation of *BraA.MET1.a* we have evidence of effects on plant stature, leaf shape and fertility (data not shown). A similar range of abnormal phenotypes is observed in Arabidopsis *ddm1* mutants [64,66]. Although developmental abnormalities were not initially reported in *B. rapa* following RNAi down-regulation of *BraA.DDM1*[67], we have observed some morphological abnormalities in subsequent generations.

The structure of the BraRoAZ population provides a means for systematic screening of epiallelic variation. The distribution of ^5m^CG marks retained in the E2 and subsequent generations is expected to be stochastic, leading to local variations in epiallelic status of adjacent genes. This offers several advantages over constitutive down-regulation of *MET1*. For example, in *met1* mutants of Arabidopsis up to 90% reduction in cytosine methylation can occur, predominantly in CpG dinucleotides [14]. Availability of epiRILs in Arabidopsis generated from a cross between wild type and the *ddm1* mutant offers an alternative strategy for characterising epigenetic effects at target loci [19]. However, this approach may not be so effective in more complex genomes such as *Brassica*, which contain multiple paralogous genic regions, and a larger load of transposable elements.

We observed variation in a range of seed yield and composition traits, within both the E2 and E3 generations. Epigenetic processes have been implicated in the regulation of seed size, with DNA methylation appearing to play a key role. For example, crosses between a hypomethylated maternal genome and wild type paternal genome lead to increased seed size, whereas the reciprocal crosses lead to reduced seed size [11,68,69]. For some lines we observed inheritance of the reduced seed size phenotype from E2 to E3 generations. We were able to demonstrate that the variation in seed size is largely independent of seed number and seed yield per plant, suggesting that there is opportunity to select and recombine specific epialleles to increase overall yield.

We found that hypomethylation had an effect across the population (E2 and E3) of increasing seed protein content, with a corresponding decrease in oil content. Given our null hypothesis was random variation around the wild-type mean for both components, this indicates that hypomethylation could lead to de-repression of specific genes that modulate the oil:protein ratio, either via specific resource allocation pathways, or through interaction with the normal seed development and maturation programme.

In addition, we have demonstrated that hypomethylation can give rise to large modulations in the proportion of key seed fatty acid components, and that these are transmitted through at least one meiotic event. These effects include decreases in erucic acid that correspond to increases in linoleic and/or palmitic acid. This level of variation is consistent with that observed in surveys of natural genetic variation and population segregation within *Brassica* species [43]. The inference of which steps in the accepted fatty acid synthesis pathways are affected allows us to postulate which sets of genes may have been up-regulated as a result of hypomethylation. This either may be due to direct effects on a specific enzyme or as a result of de-repression of an activator of a specific enzyme. For example, the reduction in the levels of erucic acid and corresponding increase in levels of linoleic acid is consistent with activation of *FAD2*[43]. The increases in palmitic acid with concomitant increase in linoleic may indicate an activation of a gene earlier in the fatty acid elongation pathways.

There was no consistent pattern of inheritance at a population level between the E2 and E3 for seed composition traits. However, this is as expected for relatively complex traits in bulked sib-seed, where there may be strong G×E interactions compounded with segregation of one or more affected loci. Moreover, greater variation was observed in the S3 controls compared with S2, underlying the importance of studying epiallelic variation in well controlled and reproducible environments. Variation in ^5m^CG marks have been implicated in modulating G×E interactions in the vernalisation response of *FLC* and *MAF-5*[70]. DNA methylation marks also tend to exclude histone H2A.Z [7], which appears to play an essential role in temperature perception [71]. As with EMS TILLING populations the stochastic distribution of (epi-)mutant alleles in early generations is likely to mask the phenotypic effect of single loci. Thus it is necessary to generate backcross lines (to wild type) through at least three generations prior to carrying out a rigorous segregation analysis and identifying the relevant loci.

Preliminary transcript analysis of leaf tissue from individual E3 plants did not show any evidence of widespread variation in gene expression, with only a small number of genes being up-regulated and none down-regulated. This is consistent with the observed phenotypic observations and de-repression of transcription expected, given the stochastic distribution of modified 5 mC sites that will have arisen from the original 5-AzaC treatment. Under controlled growth conditions on two distinct occasions, more variation was observed in transcript profile between homozygous wild type controls than was observed between two sib E3 plants grown on single occasions. More comprehensive screening of lines is required to understand the distribution and pattern of epiallelic variation affecting transcript levels within the population, and this may be amenable to targeted reverse epiallelic assays.

## Conclusions

Generating stochastically hypomethylated populations may assist epiallele discovery across a wide range of crop plants, and has considerable potential as an intervention strategy for crop improvement [3]. This will require development of strategies that ensure stable retention and deployment of desirable epialleles within breeding material or seed multiplication stocks, and also to develop new techniques for targeted epigenetic modification. These strategies need to take into account issues of re-methylation, G×E interactions, as well as epigenomic remodelling resulting from initial hybridisation events [72]. We have previously suggested [3] the need for rapid isolation of chromosomal segments carrying a modified epiallele through introgression. Targeted reverse epi-genetic screening also requires detailed knowledge of the distribution of ^5m^C in crop genomes using high throughput bisulphite sequencing [21], together with locus-specific marker assays that detect epialleles using technologies, such as high resolution melts [73].

## Methods

### Plant material and seed treatment with 5-AzaC

R-o-18 is a rapid cycling self-compatible inbred line of *B. rapa* var. *trilocularis* (Roxb.) Hanelt (yellow sarson), and was originally obtained from John Innes Centre, Norwich, UK. Seed from accession number GK070302 were treated with the demethylating agent 5-AzaCytidine (Sigma, UK), essentially as described by King [50]. A stock solution of 15 mM 5-AzaC was prepared in 50 mM 2-[N-morpholino] ethanesulphonic acid (MES) buffer at pH 6.3. To establish dose–response, five different concentrations (0.01 mM, 0.1 mM, 0.5 mM, 1.0 mM and 1.5 mM) were prepared. 50 seeds were placed evenly on filter paper within 9cm Petri dishes to which 4.5 ml 5-AzaC solution (or water, for controls) had been added, placed in a containment tray and incubated in the dark at 20°C for three days. The seeds were transferred to a sieve, washed with water at least eight times, and then sown in Rothamsted prescription mix compost in multi-celled seed trays. The trays were arranged in a randomised complete block design (RCBD) with four replicates of 100 seedlings for each of the six treatments. Unless stated otherwise, plants were grown in a glasshouse with supplementary lighting with 16 hour day length, providing a minimum of 300 μmol/m^2^/s of Photosynthetically Active Radiation. Plants were grown at nominal temperatures of 18°C day and 15°C night, with an air venting band of 2°C. However, during summer 2008 a peak temperature of 32°C was recorded, which may have affected development of the E3 population (see next section). Seedlings were transplanted into compost in 9cm pots 14 to 17 days after initial potting. In order to ensure self-pollination, inflorescences were enclosed in micro-perforated pollination bags prior to anthesis.

### Establishment of hypomethylated population BraRoAZ

“E” denotes ‘epiallelic’ by analogy with “M” for ‘mutant’. The nomenclature of seed and plant generations follows that used previously for EMS mutant populations [33]. Thus E1 plants represent those growing from seed that were imbibed with 5-AzaC. E2 is the generation after E1 and so on (Figure 2).

Sets of 250 seeds each of accession GK070302 were treated with 0.25 mM and 0.30 mM prepared from the same batch of stock 5-AzaC. The plants were grown in a RCBD which included two control sets, treated with water or with 2-[N-morpholino] ethanesulphonic acid (MES) buffer. Plants were monitored and phenotypic traits recorded during growth. Leaf material was collected from all 500 plants, freeze-dried and stored as archival material for molecular analysis. From each of 500 E1 plants three siliques were harvested and assigned separate accession numbers (total 1500). Seeds from the remaining siliques on each plant were pooled and assigned accession numbers, and total seed weight per plant recorded.

A subset of 77 lines from the two treatments was identified based on observed abnormal E1 phenotype, along with an additional 23 lines of wild-type appearance. These 100 lines were used to generate the E2 generation of plants, and were grown with six control (untreated) lines. Ten seed from each selected line (generating 1000 E2 plants, 500 per treatment) were sown in pots and arbitrarily placed within a glasshouse compartment.

BraRoAZ_E3 seeds were harvested from specified siliques from all plants and assigned unique accession numbers, totalling 1,000 lines. E1 plants which gave rise to E2 seeds were grown between October 2007 and March 2008, while E2 plants which gave rise to E3 seeds were grown between May 2008 and November 2008.

### Phenotypic trait measurements

Germination was assessed by counting the number of seeds that had germinated (by emergence of the radicle) seven days after imbibition. Plant height was recorded 20 days after sowing, with a threshold of ‘stunted’ growth set at 9cm in comparison with a minimum of 11cm among control plants. Days to first anthesis was recorded (from the date of sowing into compost). Plant survival was recorded 60 days after transplanting. Number of seeds per silique and the average seed weight of 25 seeds were recorded after harvest. Siliques were taken from nominated positions (sixth, seventh and eighth siliques, counting from the apex of the plant) of the primary inflorescence. The presence or absence of pollen was recorded. E2 germination was recorded seven days after imbibition for four replicates of 30 seed per treatment on wetted filter paper in Petri dishes. Morphological features including plant stature, floral morphology and branching were recorded in both the E1 and E2 generations.

Seed size was determined using a Marvin (Selecta Machinefabriek B.V., Enkhuizen, Netherlands) digital seed analyser with no more than 150 seeds being placed on a small seed tray. Number of seed and individual seed data were recorded using the Marvin 4.0 software, with a circularity setting of three. 100 E2 lines were sampled with 30 S2 water control lines.

Non-destructive assessment of seed composition was carried out by Near InfraRed spectroscopy (NIRS) using the NIRFlex N-500 (BUCHI UK Ltd, Oldham, UK) for approximately 200–500 seeds per line, with a 15 × 45 mm glass vial in the NIRFlex Solids Vial add-on. Two independent readings for each line were taken on different days. Samples were analysed in a randomised order on two separate days using 32 scans for each sample. An S3 MES control sample was nominated as a reference standard and analysed in alternate runs to enable normalisation of drift in measurements recorded on separate occasions. The % oil and protein is expressed on a dry matter basis whereas fatty acids are expressed as a % of total oil content. Standard fatty acid methyl esters (FAME) analysis was used to compare and validate NIRS assignments for specific components. Extraction was carried out for five seeds per vial, replicated five times for each of one wild type and three E2 lines BraRoAZ_12175e2, BraRoAZ_12175e2 and BraRoAZ_12067e2, with Heptadecanoic acid (100 mg) added as an internal standard before processing [74].

### MSAP profiling of DNA methylation in BraRoAz

#### Plant material

Ten seeds from two S2 (BraRoAZ_12845s2, BraRoAZ_12857s2), two S3 lines (BraRoAZ_12842s3, BraRoAZ_12854s3), five E2 (BraRoAZ_10263e2, BraRoAZ_10287e2, BraRoAZ_10543e2, BraRoAZ_11543e2, BraRoAZ_12447e2) and five E3 lines (BraRoAZ_10261e3, BraRoAZ_10286e3, BraRoAZ_10542e3, BraRoAZ_11541e3, BraRoAZ_12445e3) were sown in Rothamsted prescription mix compost in multi-celled seed trays and placed under controlled growth conditions (18°C day/15°C night, 16 hour day length, 300 μmol/m^2^/s of Photosynthetically Active Radiation). The first true leaf from each seedling was sampled at an equivalent developmental stage, frozen in liquid N2, stored at -80°C. The DNA was extracted using Qiagen DNeasy kit and diluted in nuclease free water to a final concentration of 10 ng/μl.

#### MSAP procedure

We used a modification of the MSAP methods described by [75] to reveal global variability in CG methylation patterns between *B. rapa* samples. For each individual, 50ng of DNA were digested and ligated for 2 h at 37°C using 5U of *EcoR*I and 1U of *Msp*I or *Hpa*II (New England Biolabs), 0.45 μM *EcoR*I adaptor, 4.5 μM *Hpa*II adaptor (see Additional file 2: Table S1 for oligonucleotide sequences) and 1U of T4 DNA ligase (Sigma) in 11 μl total volume of 1X T4 DNA ligase buffer (Sigma), 1 μl of 0.5M NaCl, supplemented with 0.5 μl at 1mg/ml of BSA. Enzymes were then inactivated by heating to 75°C for 15 min. Following restriction and adaptor ligation there followed two successive rounds of PCR amplification. For preselective amplification, 0.3 μl of the restriction/ligation products described above were incubated in 12.5 μl volumes containing 1X Biomix (Bioline, London, UK) with 0.05 μl of Preamp*EcoR*I primer and 0.25 μl Preamp*HpaII*/*Msp*I (both primers at 10 uM) (see Additional file 2: Table S1 primers sequences) supplemented with 0.1 μl at 1mg/ml of BSA. PCR conditions were 2 min at 72_C followed by 30 cycles of 94_C for 30 s, 56_C for 30 s and 72_C for 2 min with a final extension step of 10 min at 72°C. Selective PCR reactions were performed using 0.3 μl of preselective PCR reaction product and the same reagents as the preselective reactions but using FAM labeled selective primers (E2/H1 or E3/H3; see Additional file 2: Table S1 for primer sequence). Cycling conditions for selective PCR were as follows: 94°C for 2 min, 13 cycles of 94°C for 30 s, 65°C (decreasing by 0.7°C each cycle) for 30 s, and 72°C for 2 min, followed by 24 cycles of 94°C for 30 s, 56°C for 30 s, and 72°C for 2min, ending with 72°C for 10 min.

Fluorescently labeled MSAP products were diluted 1:10 in nanopure sterile water and 1 μl was combined with 1 μl of ROX/HiDi mix (50 μl ROX plus 1 ml of HiDi formamide, Applied Biosystems, USA). Samples were heat-denatured at 95°C for 3–5 min and snap-cooled on ice for 2 min. Samples were fractionated on an ABI PRISM 3100 at 3 kV for 22 s and at 15 kV for 45 min.

### Statistical Analysis

#### Analysis of morphological variance

GenStat® (2010, 13^th^ edition, VSN International Ltd, Hemel Hempstead, UK) statistical package was used for all analyses. ANOVA was used to analyse trait variation and the significance of differences between the 5-AzaC concentrations was determined using the F-test, and least significant difference (LSD) at the 5% level was used to compare means [76]. Analysis of residuals indicated no requirement for transformation of the data. A logistic curve modified to start at 100% (survived or normal plants) was used to model the dose response data. The model was fitted using nonlinear least squares regression, to estimate the three possible parameters (the exponential rate, the dose giving 50% survived or normal plants and the lower asymptote) [76]. F-test was used to assess whether a lower asymptote was required, and to assess lack-of-fit of the model. Restricted Maximum Likelihood (REML, [77] Section 5.3.3) analysis was used to estimate variation within and between populations of treated and control lines of the same generation. Differences between populations in seed area were assessed using Wald tests ([77]Section 6.2) and differences in population variances were assessed using likelihood ratio tests ([77], Section 6.3).

#### Analysis of epigenetic variance

Epigenetic similarity between tested samples was determined by Principal Coordinate Analysis (PCoA) [78] based on the MSAP profiles obtained from primer combinations E2/H1 and E3/H3 using GenAlex (v.6.4). We then used Analysis of Molecular Variance (AMOVA) [79] to evaluate the structure and degree of epigenetic diversity induced by 5-AzaC. Pairwise PhiPT [80] comparisons (an analogue of the F_st_ fixation index, that measures differential connectivity/genetic diversity among populations) between samples from a line restricted with *Hpa*II or *Msp*I was used to infer their overall level of divergence in DNA methylation within the targeted restriction sites (i.e., the lower the PhiPT value between samples restricted using *Hpa*II and the same samples restricted with *Msp*I, the lower the level of DNA methylation in those samples). AMOVA was subsequently calculated using GenAlex (v.6.4) to test the significance of PhiPT between populations [80], with the probability of non-differentiation (FST=0) being estimated over 9,999 permutations. The sum of squares within population SSWP [80] was thereby used to infer epigenetic variation within lines.

#### Cytological localisation of ^5m^C by immune-staining

Four plants from two E2 lines and an S2 water control line were grown in controlled environment conditions of 18–15°C, 16 hour daylength and relative humidity of 65 -75%. Immature flower buds (1.2mm) were sampled and fixed as described by [42] to obtain pachytene stage chromosomes from meiotic pollen mother cells. Detection of ^5m^C was performed as described in [47] using mouse monoclonal anti-^5m^C antibody (Calbiochem, UK).

#### Transcriptome analysis

Total RNA was extracted using Qiagen RNeasy plant kit from leaf 6 of three separate plants of E3 line BraRoAZ_12445e3 (phenotypically indistinguishable from control, with small seeds), and two separate plants of R-o-18. Labelling and hybridisation to the Affymetrix GeneChip Brassica Exon 1.0 ST Array [40] was carried out by the NASCArrays service (http://www.arabidopsis.info). Results were analysed using GeneSpring v.11.5, with GO analysis essentially following that described by [44].

Seed of the BraRoAZ_E2 and BraRoAZ_E3 populations are available from smita.kurup@rothamsted.ac.uk. Re-sequencing genomic data for R-o-18 are available online at http://www.brassica.info/datasets/Brassica_resequencing_data/.

## Competing interests

The authors declare that they have no competing interests.

## Authors’ contributions

SA generated the population and analysed seed traits, SK assisted with population generation, measurement of traits, generation of plants for MSAP, and drafting the manuscript. CL carried out the MSAP analyses. SJW and SJP performed the statistical analyses. CH assisted with population generation. MW participated in the design of the MSAP analyses and drafting the manuscript. GK conceived of the study, participated in its design and coordination and drafting the manuscript. All authors read and approved the final manuscript.

## Supplementary Material

Additional file 1 **Figure S1.** Dosage response curve for seedling growth of B. rapa R-o-18 raised from seeds pre-treated with 5-AzaC. B. rapa seeds were treated with five different concentrations of 5-AzaC (0.01mM, 0.1mM, 0.5mM, 1.0mM and 1.5mM) and a control. A gradient of retarded growth was observed with increasing concentration. This was quantified by measuring the height of individual plants (Figure 1a). **Figure S2.** Effect of 5-AzaC treatments on flowering time in B. rapa R-o-18. Seeds were treated with five different concentrations of 5-AzaC (0.01mM, 0.1mM, 0.5mM, 1.0mM and 1.5mM) and a water control. Days to flowering, defined as the number of days that had lapsed since seeds were sown in soil to the day the first anthesis was observed on the plant. Data points in graph represent the mean number of days to flowering at the concentrations studied. **Figure S3.** Box-plot analysis of E2 seed weight resulting from 5-AzaC treatment. Seed were harvested from plants derived from seeds that had been treated with 5-AzaC. At lower concentrations of 5-AzaC some E1 plants had heavier seeds and others with lighter seeds. The box represents 50% of plants. The whiskers at the top and the bottom of the boxes represent the upper and the lower quartiles respectively. The horizontal line across the boxes indicates the median seed weight. Click here for file

Additional file 2 **Table S1.** Primers used for MSAP analysis. Selective nucleotides are indicated as +XYZ in the primer code column. Enzyme column indicates the restriction enzyme site associate with each primer. **Table S2.** Epigenetic molecular diversity induced by 5-AzaC. Epigenetic diversity induced by 5-AzaC calculated using Analysis of Molecular Variance (AMOVA) inferred from the analysis of methylation-sensitive amplified polymorphism (MSAP) assays using primer combinations H2/E1 and H3/E3. Populations are ordered following their PhiPT values which indicate the epigenetic distance between each population restricted with HpaII and MspI (Populations highlighted in red presented significantly lower PhiPTs when compared to the original *B. rapa* line R-o-18). Prob indicates the probability of having a more extreme variance component and PhiPT than the observed values by chance alone. The Sum of Squares within population (**SSWP**) reflects intra-population diversity from the analysis of methylation-sensitive amplified polymorphism (MSAP) assays using the methylation sensitive restriction enzyme *Hpa*II. Click here for file

Additional file 3 **Table S3.** Genes identified as upregulated in Brassica rapa line E3 line BraRoAZ_12445e3 compared with the R-o-18 control line. Leaf RNA was hybridised against the GeneChip Brassica Exon 1.0 ST Array [3940]. Results were analysed using GeneSpring v.11.5, with GO analysis essentially following that described by Love et al. [40]. Click here for file

## References

[B1] Manning K Tör M Poole M Hong Y Thompson AJ King GJ Giovannoni JJ Seymour GB A naturally occurring epigenetic mutation in a gene encoding an SBP-box transcription factor inhibits tomato fruit ripening Nat Genet 2006 38 8 948 952 16832354 10.1038/ng1841

[B2] Kalisz S Purugganan MD Epialleles via DNA methylation: consequences for plant evolution Trends Ecol Evol 2004 19 6 309 314 16701276 10.1016/j.tree.2004.03.034

[B3] King GJ Amoah S Kurup S Exploring and exploiting epigenetic variation in crops Genome 2010 53 11 856 868 21076501 10.1139/G10-059

[B4] Hauben M Haesendonckx B Standaert E Van Der Kelen K Azmi A Akpo H Van Breusegem F Guisez Y Bots M Lambert B Energy use efficiency is characterized by an epigenetic component that can be directed through artificial selection to increase yield Proc Natl Acad Sci 2009 106 47 20109 19897729 10.1073/pnas.0908755106 PMC2774259

[B5] Smith AP Jain A Deal RB Nagarajan VK Poling MD Raghothama KG Meagher RB Histone H2A. Z regulates the expression of several classes of phosphate starvation response genes but not as a transcriptional activator Plant Physiol 2010 152 1 217 19897606 10.1104/pp.109.145532 PMC2799358

[B6] Kim M Ohr H Lee JW Hyun Y Fischer RL Choi Y Temporal and spatial downregulation of Arabidopsis MET1 activity results in global DNA hypomethylation and developmental defects Mol Cells 2008 26 611 615 18820427 PMC4109710

[B7] Zilberman D Coleman-Derr D Ballinger T Henikoff S Histone H2A. Z and DNA methylation are mutually antagonistic chromatin marks Nature 2008 456 7218 125 129 18815594 10.1038/nature07324 PMC2877514

[B8] Shiba H Kakizaki T Iwano M Tarutani Y Watanabe M Isogai A Takayama S Dominance relationships between self-incompatibility alleles controlled by DNA methylation Nat Genet 2006 38 3 297 299 16444272 10.1038/ng1734

[B9] Dennis E Finnegan E Bilodeau P Chaudhury A Genger R Helliwell C Sheldon C Bagnall D Peacock W Vernalization and the initiation of flowering Semin Cell Dev Biol 1996 7 3 441 448

[B10] Kondo H Miura T Wada KC Takeno K Induction of flowering by 5 azacytidine in some plant species: relationship between the stability of photoperiodically induced flowering and flower inducing effect of DNA demethylation Physiol Plant 2007 131 3 462 469 18251884 10.1111/j.1399-3054.2007.00965.x

[B11] FitzGerald J Luo M Chaudhury A Berger F DNA methylation causes predominant maternal controls of plant embryo growth PLoS One 2008 3 5 2298 10.1371/journal.pone.0002298 PMC2390113 18509545

[B12] Gehring M Bubb KL Henikoff S Extensive demethylation of repetitive elements during seed development underlies gene imprinting Science 2009 324 5933 1447 19520961 10.1126/science.1171609 PMC2886585

[B13] Finnegan EJ Dennis ES Isolation and identification by sequence homology of a putative cytosine methyltransferase from Arabidopsis thaliana Nucleic Acids Res 1993 21 10 2383 8389441 10.1093/nar/21.10.2383 PMC309536

[B14] Finnegan EJ Peacock WJ Dennis ES Reduced DNA methylation in Arabidopsis thaliana results in abnormal plant development Proc Natl Acad Sci U S A 1996 93 16 8449 8710891 10.1073/pnas.93.16.8449 PMC38691

[B15] Brzeski J Jerzmanowski A Deficient in DNA Methylation 1 (DDM1) Defines a Novel Family of Chromatin-remodeling Factors J Biol Chem 2003 278 2 823 828 12403775 10.1074/jbc.M209260200

[B16] Martienssen RA Colot V DNA methylation and epigenetic inheritance in plants and filamentous fungi Science 2001 293 5532 1070 11498574 10.1126/science.293.5532.1070

[B17] Saze H Epigenetic memory transmission through mitosis and meiosis in plants Semin Cell Dev Biol 2008 19 6 527 536 18708156 10.1016/j.semcdb.2008.07.017

[B18] Long Y Xia W Li R Wang J Shao M Feng J King GJ Meng J Epigenetic QTL mapping in Brassica napus Genetics 2011 189 3 1093 1102 21890742 10.1534/genetics.111.131615 PMC3213370

[B19] Johannes F Porcher E Teixeira FK Saliba-Colombani V Simon M Agier N Bulski A Albuisson J Heredia F Audigier P Bouchez D Dillmann C Guerche P Hospital F Colot V Assessing the impact of transgenerational epigenetic variation on complex traits PLoS Genet 2009 5 6 e1000530 10.1371/journal.pgen.1000530 19557164 PMC2696037

[B20] Roux F Colomé-Tatché M Edelist C Wardenaar R Guerche P Hos-pital F Colot V Jansen RC Johannes F Genome-wideepigenetic perturbation jump-starts patterns of heritable variation found in nature Genetics 2011 188 1015 1017 21596900 10.1534/genetics.111.128744 PMC3176100

[B21] Cokus SJ Feng S Zhang X Chen Z Merriman B Haudenschild CD Pradhan S Nelson SF Pellegrini M Jacobsen SE Shotgun bisulphite sequencing of the Arabidopsis genome reveals DNA methylation patterning Nature 2008 452 7184 215 219 18278030 10.1038/nature06745 PMC2377394

[B22] Lister R O’Malley RC Tonti-Filippini J Gregory BD Berry CC Millar AH Ecker JR Highly integrated single-base resolution maps of the epigenome in Arabidopsis Cell 2008 133 3 523 536 18423832 10.1016/j.cell.2008.03.029 PMC2723732

[B23] Chodavarapu RK Feng S Bernatavichute YV Chen PY Stroud H Yu Y Hetzel JA Kuo F Kim J Cokus SJ Relationship between nucleosome positioning and DNA methylation Nature 2010 466 7304 388 392 20512117 10.1038/nature09147 PMC2964354

[B24] Bossdorf O Arcuri D Richards CL Pigliucci M Experimental alteration of DNA methylation affects the phenotypic plasticity of ecologically relevant traits in Arabidopsis thaliana Evolutionary Ecology 2010 24 541 553

[B25] Johannes F Colomé-Tatché M Quantitative epigenetics through epigenomic perturbation of isogenic lines Genetics 2011 188 215 227 21385727 10.1534/genetics.111.127118 PMC3120148

[B26] Christman JK 5-Azacytidine and 5-aza-2’-deoxycytidine as inhibitors of DNA methylation: mechanistic studies and their implications for cancer therapy Oncogene 2002 21 35 5483 5495 12154409 10.1038/sj.onc.1205699

[B27] Šorm F Piskala A Čihák A Veselý J 5-Azacytidine, a new, highly effective cancerostatic Cell Mol Life Sci 1964 20 4 202 203 10.1007/BF02135399 5322617

[B28] Lyko F Brown R DNA methyltransferase inhibitors and the development of epigenetic cancer therapies J Natl Cancer Inst 2005 97 20 1498 16234563 10.1093/jnci/dji311

[B29] Santi DV Garrett CE Barr PJ On the mechanism of inhibition of DNA-cytosine methyltransferases by cytosine analogs Cell 1983 33 1 9 6205762 10.1016/0092-8674(83)90327-6

[B30] Santi DV Norment A Garrett CE Covalent bond formation between a DNA-cytosine methyltransferase and DNA containing 5-azacytosine Proc Natl Acad Sci U S A 1984 81 22 6993 6209710 10.1073/pnas.81.22.6993 PMC392062

[B31] Stresemann C Brueckner B Musch T Stopper H Lyko F Functional diversity of DNA methyltransferase inhibitors in human cancer cell lines Cancer Res 2006 66 5 2794 16510601 10.1158/0008-5472.CAN-05-2821

[B32] Eichten SR Swanson-Wagner RA Schnable JC Waters AJ Hermanson PJ Liu S Yeh C-T Freeling M Schnable PS Vaughn MW Springer NM Heritable epigenetic variation amongst maize inbreds PLoS Genet 2011 7 e1002372 22125494 10.1371/journal.pgen.1002372 PMC3219600

[B33] Stephenson P Baker D Girin T Perez A Amoah S King GJ Østergaard L A rich TILLING resource for studying gene function in Brassica rapa BMC Plant Biol 2010 10 1 62 20380715 10.1186/1471-2229-10-62 PMC2923536

[B34] Wang N Wang Y Tian F King GJ Zhang C Long Y Shi L Meng J A functional genomics resource for Brassica napus: development of an EMS mutagenized population and discovery of FAE1 point mutations by TILLING New Phytol 2008 180 4 751 765 18811617 10.1111/j.1469-8137.2008.02619.x

[B35] Uauy C Paraiso F Colasuonno P Tran RK Tsai H Berardi S Comai L Dubcovsky J A modified TILLING approach to detect induced mutations in tetraploid and hexaploid wheat BMC Plant Biol 2009 9 1 115 19712486 10.1186/1471-2229-9-115 PMC2748083

[B36] Gómez-Campo C Biology of Brassica coenospecies 1999 Amsterdam, The Netherlands: Elsevier Science Ltd

[B37] Lukens LN Pires JC Leon E Vogelzang R Oslach L Osborn T Patterns of sequence loss and cytosine methylation within a population of newly resynthesized Brassica napus allopolyploids Plant Physiol 2006 140 1 336 16377753 10.1104/pp.105.066308 PMC1326055

[B38] Lukens LN Quijada PA Udall J Pires JC Schranz ME Osborn TC Genome redundancy and plasticity within ancient and recent Brassica crop species Biol J Linn Soc 2004 82 4 665 674

[B39] Xu Y Zhong L Wu X Fang X Wang J Rapid alterations of gene expression and cytosine methylation in newly synthesized Brassica napus allopolyploids Planta 2009 229 3 471 483 18998158 10.1007/s00425-008-0844-8

[B40] Salmon A Clotault J Jenczewski E Chable V Manzanares-Dauleux MJ Brassica oleracea displays a high level of DNA methylation polymorphism Plant Sci 2008 174 1 61 70

[B41] Glyn MCP Egertová M Gazdova B Kovarik A Bezdek M Leitch AR The influence of 5-azacytidine on the condensation of the short arm of rye chromosome 1R in Triticum aestivum L. root tip meristematic nuclei Chromosoma 1997 106 8 485 492 9426280 10.1007/pl00007688

[B42] Lu G Wu X Chen B Gao G Xu K Li X Detection of DNA methylation changes during seed germination in rapeseed (Brassica napus) Chinese Science Bulletin 2006 51 2 182 190

[B43] Barker GC Larson TR Graham IA Lynn JR King GJ Novel insights into seed fatty acid synthesis and modification pathways from genetic diversity and quantitative trait Loci analysis of the Brassica C Genome Plant Physiol 2007 144 4 1827 1842 17573542 10.1104/pp.107.096172 PMC1949901

[B44] Love CG Graham NS Lochlainn SÓ Bowen HC May ST White PJ Broadley MR Hammond JP King GJ A Brassica Exon Array for whole-transcript gene expression profiling PLoS One 2010 5 9 e12812 10.1371/journal.pone.0012812 20862292 PMC2940909

[B45] Till BJ Reynolds SH Greene EA Codomo CA Enns LC Johnson JE Burtner C Odden AR Young K Taylor NE Large-scale discovery of induced point mutations with high-throughput TILLING Genome Res 2003 13 3 524 12618384 10.1101/gr.977903 PMC430291

[B46] Haaf T The effects of 5-azacytidine and 5-azadeoxycytidine on chromosome structure and function: implications for methylation-associated cellular processes Pharmacol Ther 1995 65 1 19 46 7536332 10.1016/0163-7258(94)00053-6

[B47] Castilho A Neves N Rufini-Castiglione M Viegas W Heslop-Harrison J 5-Methylcytosine distribution and genome organization in triticale before and after treatment with 5-azacytidine J Cell Sci 1999 112 23 4397 4404 10564657 10.1242/jcs.112.23.4397

[B48] Brown JCL De Decker MM Fieldes MA A comparative analysis of developmental profiles for DNA methylation in 5-azacytidine-induced early-flowering flax lines and their control Plant Sci 2008 175 3 217 225

[B49] Kondo H Ozaki H Itoh K Kato A Takeno K Flowering induced by 5 azacytidine, a DNA demethylating reagent in a short day plant. Perilla frutescens var. crispa Physiol Plant 2006 127 1 130 137

[B50] King G Morphological development in Brassica oleracea is modulated by in vivo treatment with 5-azacytidine Journal of Horticultural Science (United Kingdom) 1995 70 333 342

[B51] Johnston JS Pepper AE Hall AE Chen ZJ Hodnett G Drabek J Lopez R Price HJ Evolution of genome size in Brassicaceae Ann Bot 2005 95 1 229 15596470 10.1093/aob/mci016 PMC1950721

[B52] Alix K Joets J Ryder CD Moore J Barker GC Bailey JP King GJ Heslop-Harrison JS The CACTA transposon Bot1 played a major role in Brassica genome divergence and gene proliferation Plant J 2008 56 6 1030 1044 18764926 10.1111/j.1365-313X.2008.03660.x

[B53] Braszewska-Zalewska A Bernas T Maluszynska J Epigenetic chromatin modifications in Brassica genomes Genome 2010 53 203 210 20237597 10.1139/g09-088

[B54] Koornneef M Alonso-Blanco C Peeters AJM Soppe W Genetic control of flowering time in Arabidopsis Annu Rev Plant Biol 1998 49 1 345 370 10.1146/annurev.arplant.49.1.345 15012238

[B55] Mouradov A Cremer F Coupland G Control of flowering time: interacting pathways as a basis for diversity The Plant Cell Online 2002 14 90001 111 10.1105/tpc.001362 PMC151251 12045273

[B56] Putterill J Laurie R Macknight R It’s time to flower: the genetic control of flowering time Bioessays 2004 26 4 363 373 15057934 10.1002/bies.20021

[B57] Genger RK Peacock JW Dennis ES Finnegan JE Opposing effects of reduced DNA methylation on flowering time in Arabidopsis thaliana Planta 2003 216 3 461 466 12520338 10.1007/s00425-002-0855-9

[B58] Jullien PE Kinoshita T Ohad N Berger F Maintenance of DNA methylation during the Arabidopsis life cycle is essential for parental imprinting Plant Cell 2006 18 6 1360 1372 16648367 10.1105/tpc.106.041178 PMC1475502

[B59] Kinoshita T Jullien J Gurdon JB Two-dimensional morphogen gradient in Xenopus: boundary formation and real-time transduction response Dev Dyn 2006 235 12 3189 3198 17029288 10.1002/dvdy.20963

[B60] Soppe WJ Jacobsen SE Alonso-Blanco C Jackson JP Kakutani T Koornneef M Peeters AJ The late flowering phenotype of fwa mutants is caused by gain-of-function epigenetic alleles of a homeodomain gene Mol Cell 2000 6 4 791 802 11090618 10.1016/s1097-2765(05)00090-0

[B61] Burn JE Bagnall DJ Metzger JD Dennis ES Peacock WJ DNA methylation, vernalization, and the initiation of flowering Proc Natl Acad Sci U S A 1993 90 1 287 291 11607346 10.1073/pnas.90.1.287 PMC45645

[B62] Henderson IR Jacobsen SE Epigenetic inheritance in plants Nature 2007 447 7143 418 424 17522675 10.1038/nature05917

[B63] Takeda S Paszkowski J DNA methylation and epigenetic inheritance during plant gametogenesis Chromosoma 2006 115 1 27 35 16249938 10.1007/s00412-005-0031-7

[B64] Kakutani T Jeddeloh JA Flowers SK Munakata K Richards EJ Developmental abnormalities and epimutations associated with DNA hypomethylation mutations Proc Natl Acad Sci U S A 1996 93 22 12406 8901594 10.1073/pnas.93.22.12406 PMC38004

[B65] Kankel MW Ramsey DE Stokes TL Flowers SK Haag JR Jeddeloh JA Riddle NC Verbsky ML Richards EJ Arabidopsis MET1 cytosine methyltransferase mutants Genetics 2003 163 3 1109 12663548 10.1093/genetics/163.3.1109 PMC1462485

[B66] Kakutani T Jeddeloh JA Richards EJ Characterization of an Arabidopsis thaliana DNA hypomethylation mutant Nucleic Acids Res 1995 23 1 130 7870578 10.1093/nar/23.1.130 PMC306640

[B67] Fujimoto R Sasaki T Inoue H Nishio T Hypomethylation and transcriptional reactivation of retrotransposon-like sequences in ddm1 transgenic plants of Brassica rapa Plant Mol Biol 2008 66 5 463 473 18236011 10.1007/s11103-007-9285-1

[B68] Adams S Vinkenoog R Spielman M Dickinson HG Scott RJ Parent-of-origin effects on seed development in Arabidopsis thaliana require DNA methylation Development 2000 127 11 2493 2502 10804189 10.1242/dev.127.11.2493

[B69] Xiao W Brown RC Lemmon BE Harada JJ Goldberg RB Fischer RL Regulation of seed size by hypomethylation of maternal and paternal genomes Plant Physiol 2006 142 3 1160 17012404 10.1104/pp.106.088849 PMC1630758

[B70] Finnegan EJ Sheldon CC Jardinaud F Peacock WJ Dennis ES A cluster of Arabidopsis genes with a coordinate response to an environmental stimulus Curr Biol 2004 14 10 911 916 15186749 10.1016/j.cub.2004.04.045

[B71] Kumar SV Wigge PA H2A.Z-containing nucleosomes mediate the thermosensory response in Arabidopsis Cell 2010 140 1 136 147 20079334 10.1016/j.cell.2009.11.006

[B72] Michalak P Epigenetic, transposon and small RNA determinants of hybrid dysfunctions Heredity 2008 102 1 45 50 18545265 10.1038/hdy.2008.48

[B73] Wojdacz TK Dobrovic A Methylation-sensitive high resolution melting (MS-HRM): a new approach for sensitive and high-throughput assessment of methylation Nucleic Acids Res 2007 35 6 e41 17289753 10.1093/nar/gkm013 PMC1874596

[B74] Beaudoin F Wu X Li F Haslam RP Markham JE Zheng H Napier JA Kunst L Functional characterization of the Arabidopsis {beta}-Ketoacyl-Coenzyme a reductase candidates of the fatty acid Elongase Plant Physiol 2009 150 3 1174 1191 19439572 10.1104/pp.109.137497 PMC2705042

[B75] Reyna-Lopez GE Simpson J Ruiz-Herrera J Differences in DNA methylation patterns are detectable during the dimorphic transition of fungi by amplification of restriction polymorphisms Mol Gen Genet 1997 253 6 703 710 9079881 10.1007/s004380050374

[B76] Mead R, Curnow RN, Hasted AM Statistical methods in agriculture and experimental biology 2002 Boca Raton, Florida, USA: CRC Press Inc

[B77] Verbeke G, Molenberghs G Linear Mixed Models for Longitudinal Data 2000 2 New York, USA: Springer Verlag

[B78] Gower JC Some distance properties of latent root and vector methods used in multivariate analysis Biometrika 1966 53 3–4 325 338

[B79] Excoffier L Smouse PE Quattro JM Analysis of molecular variance inferred from metric distances among DNA haplotypes: application to human mitochondrial DNA restriction data Genetics 1992 131 2 479 491 1644282 10.1093/genetics/131.2.479 PMC1205020

[B80] Michalakis Y Excoffier L A generic estimation of population subdivision using distances between alleles with special reference for microsatellite loci Genetics 1996 142 1061 1064 8849912 10.1093/genetics/142.3.1061 PMC1207006

