# Supplementary material for: A Hypomethylated population of Brassica rapa for forward and reverse Epi-genetics
Source: BMC Plant Biol. 2012 Oct 20;12:193. doi: 10.1186/1471-2229-12-193 (PMC3507869; doi:10.1186/1471-2229-12-193)
Supplement: Additional file 1 — Figure S1. Dosage response curve for seedling growth of B. rapa R-o-18 raised from seeds pre-treated with 5-AzaC. B. rapa seeds were treated with five different concentrations of 5-AzaC (0.01mM, 0.1mM, 0.5mM, 1.0mM and 1.5mM) and a control. A gradient of retarded growth was observed with increasing concentration. This was quantified by measuring the height of individual plants (Figure 1a). Figure S2. Effect of 5-AzaC treatments on flowering time in B. rapa R-o-18. Seeds were treated with five different concentrations of 5-AzaC (0.01mM, 0.1mM, 0.5mM, 1.0mM and 1.5mM) and a water control. Days to flowering, defined as the number of days that had lapsed since seeds were sown in soil to the day the first anthesis was observed on the plant. Data points in graph represent the mean number of days to flowering at the concentrations studied. Figure S3. Box-plot analysis of E2 seed weight resulting from 5-AzaC treatment. Seed were harvested from plants derived from seeds that had been treated with 5-AzaC. At lower concentrations of 5-AzaC some E1 plants had heavier seeds and others with lighter seeds. The box represents 50% of plants. The whiskers at the top and the bottom of the boxes represent the upper and the lower quartiles respectively. The horizontal line across the boxes indicates the median seed weight. [file 1471-2229-12-193-S1.docx]

**Figure S1**. Dosage response curve for **seedling growth** of *B. rapa* R-o-18 raised from seeds pre-treated with 5-AzaC. *B. rapa* seeds were treated with five different concentrations of 5-AzaC (0.01mM, 0.1mM, 0.5mM, 1.0mM and 1.5mM) and a control. A gradient of retarded growth was observed with increasing concentration. This was quantified by measuring the height of individual plants (Fig. 1a).

**
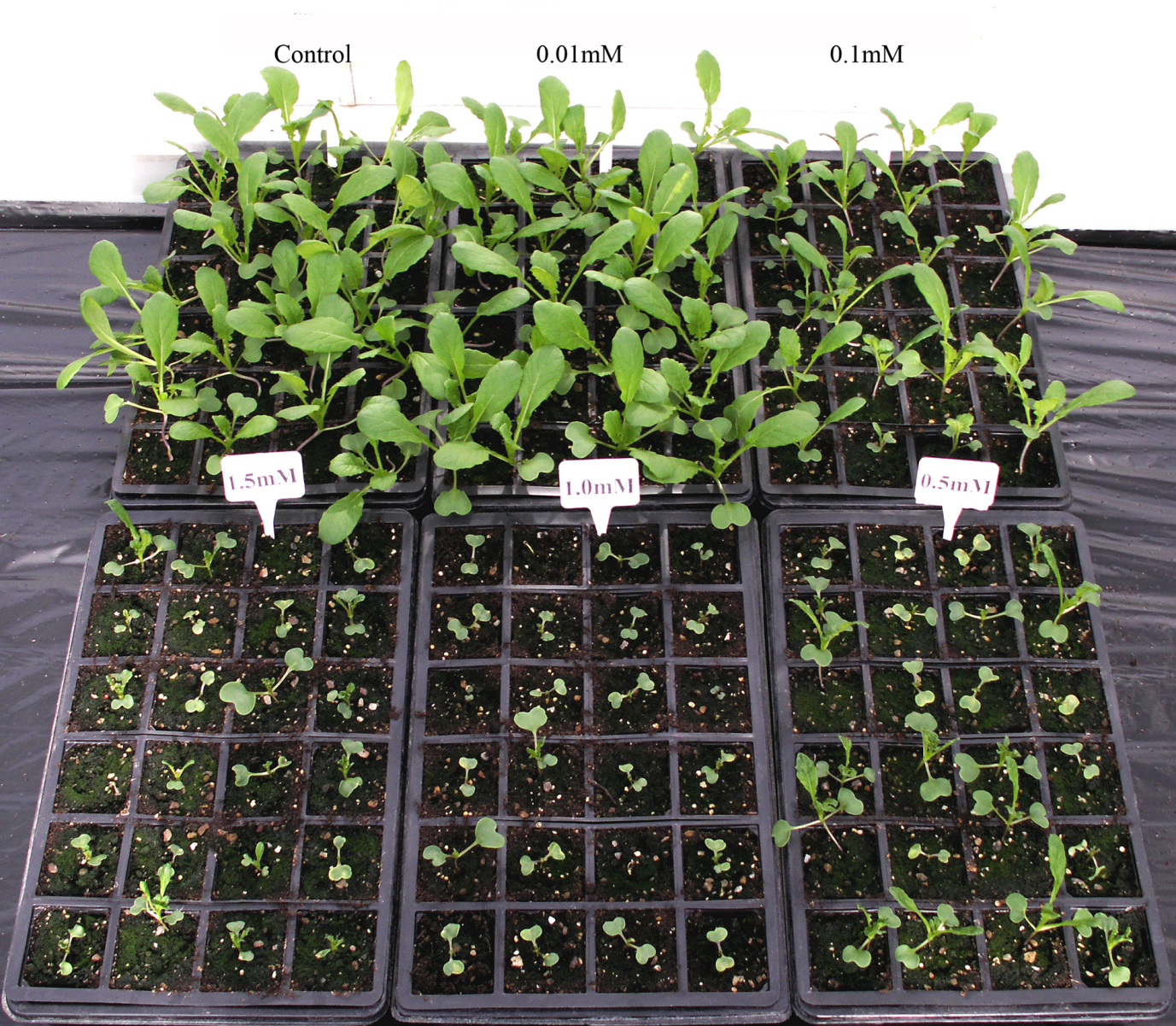
S1**

**l.s.d**

**Figure S2**. Effect of 5-AzaC treatments on **flowering time** in *B. rapa* R-o-18*.* Seeds were treated with five different concentrations of 5-AzaC (0.01mM, 0.1mM, 0.5mM, 1.0mM and 1.5mM) and a water control. Days to flowering, defined as the number of days that had lapsed since seeds were sown in soil to the day the first anthesis was observed on the plant. Data points in graph represent the mean number of days to flowering at the concentrations studied.

**Figure S3**. Box-plot analysis of E2 seed weight resulting from 5-AzaC treatment. Seed were harvested from plants derived from seeds that had been treated with 5-AzaC. At lower concentrations of 5-AzaC some E1 plants had heavier seeds and others with lighter seeds. The box represents 50% of plants. The whiskers at the top and the bottom of the boxes represent the upper and the lower quartiles respectively. The horizontal line across the boxes indicates the median seed weight.
