# Supplementary material for: A Hypomethylated population of Brassica rapa for forward and reverse Epi-genetics
Source: BMC Plant Biol. 2012 Oct 20;12:193. doi: 10.1186/1471-2229-12-193 (PMC3507869; doi:10.1186/1471-2229-12-193)
Supplement: Additional file 2 — Table S1. Primers used for MSAP analysis. Selective nucleotides are indicated as +XYZ in the primer code column. Enzyme column indicates the restriction enzyme site associate with each primer. Table S2. Epigenetic molecular diversity induced by 5-AzaC. Epigenetic diversity induced by 5-AzaC calculated using Analysis of Molecular Variance (AMOVA) inferred from the analysis of methylation-sensitive amplified polymorphism (MSAP) assays using primer combinations H2/E1 and H3/E3. Populations are ordered following their PhiPT values which indicate the epigenetic distance between each population restricted with HpaII and MspI (Populations highlighted in red presented significantly lower PhiPTs when compared to the original B. rapa line R-o-18). Prob indicates the probability of having a more extreme variance component and PhiPT than the observed values by chance alone. The Sum of Squares within population (SSWP) reflects intra-population diversity from the analysis of methylation-sensitive amplified polymorphism (MSAP) assays using the methylation sensitive restriction enzyme HpaII. [file 1471-2229-12-193-S2.docx]

**Table S1**. Primers used for MSAP analysis. Selective nucleotides are indicated as +XYZ in the primer code column. Enzyme column indicates the restriction enzyme site associate with each primer.

| **Primer code^a^** | **Sequence** | **Enzyme** |
| --- | --- | --- |
| E2 (+AAC) | GACTGCGTACCAATTCAAC | *Eco*RI |
| E3 (+AAG) | GACTGCGTACCAATTCAAG | *Eco*RI |
| H1 (+CA) | GATGAGTCCTGAGCGGCA | *Hpa*II/*Msp*I |
| H2 (+CC) | GATGAGTCCTGAGCGGCC | *Hpa*II/*Msp*I |

**Table S2. Epigenetic molecular diversity induced by 5-AzaC**

Epigenetic diversity induced by 5-AzaC calculated using Analysis of Molecular Variance (AMOVA) inferred from the analysis of methylation-sensitive amplified polymorphism (MSAP) assays using primer combinations H2/E1 and H3/E3. Populations are ordered following their PhiPT values which indicate the epigenetic distance between each population restricted with HpaII and MspI (Populations highlighted in red presented significantly lower PhiPTs when compared to the original *B. rapa* line R-o-18). Prob indicates the probability of having a more extreme variance component and PhiPT than the observed values by chance alone. The Sum of Squares within population (**SSWP**) reflects intra-population diversity from the analysis of methylation-sensitive amplified polymorphism (MSAP) assays using the methylation sensitive restriction enzyme *Hpa*II.

| **E2H1** | | | | | **E3H3** | | | | |
| --- | --- | --- | --- | --- | --- | --- | --- | --- | --- |
| **Population** | **PhiPT** | **Prob** | **SSWP** | | **Population** | **PhiPT** | **Prob** | **SSWP** | |
|  |  |  | **HpaII** | **MspI** |  |  |  | **HpaII** | **MspI** |
| R-o-18 | 0,803 | 0,0317 | 9,00 | 11,00 | R-o-18 | 0,689 | 0,0270 | 16,00 | 6,75 |
| BraRoAZ_12842s3 | 0,785 | 0,0001 | 25,10 | 37,10 | BraRoAZ_12445e3 | 0,680 | 0,0001 | 32,00 | 27,60 |
| BraRoAZ_10287e2 | 0,781 | 0,0001 | 30,40 | 27,60 | BraRoAZ_12845s2 | 0,675 | 0,0001 | 22,80 | 36,70 |
| BraRoAZ_12445e3 | 0,778 | 0,0001 | 26,60 | 27,10 | BraRoAZ_11541e3 | 0,662 | 0,0001 | 36,10 | 29,50 |
| BraRoAZ_10286e3 | 0,751 | 0,0001 | 42,00 | 30,70 | BraRoAZ_10287e2 | 0,655 | 0,0001 | 34,80 | 26,90 |
| BraRoAZ_11541e3 | 0,735 | 0,0001 | 29,20 | 39,50 | BraRoAZ_12842s3 | 0,642 | 0,0001 | 47,40 | 33,50 |
| BraRoAZ_10543e2 | 0,731 | 0,0001 | 41,70 | 33,30 | BraRoAZ_10263e2 | 0,632 | 0,0001 | 44,00 | 32,20 |
| BraRoAZ_10263e2 | 0,728 | 0,0001 | 36,40 | 38,90 | BraRoAZ_10543e2 | 0,624 | 0,0001 | 40,20 | 31,50 |
| BraRoAZ_12854s3 | 0,721 | 0,0001 | 39,10 | 45,20 | BraRoAZ_10286e3 | 0,595 | 0,0001 | 47,10 | 24,00 |
| BraRoAZ_12857s2 | 0,651 | 0,0001 | 53,80 | 43,80 | BraRoAZ_12854s3 | 0,564 | 0,0001 | 49,20 | 41,90 |
| BraRoAZ_12845e2 | 0,628 | 0,0001 | 40,90 | 53,20 | BraRoAZ_12857s2 | 0,526 | 0,0001 | 58,00 | 38,90 |
| **BraRoAZ_11543e2** | 0,165 | 0,0014 | 133,10 | 162,20 | **BraRoAZ_11543e2** | 0,123 | 0,0192 | 88,70 | 114,10 |
| **BraRoAZ_12447e2** | 0,088 | 0,0006 | 152,10 | 150,90 | **BraRoAZ_10261e3** | 0,113 | 0,0092 | 70,20 | 107,70 |
| **BraRoAZ_10261e3** | 0,086 | 0,0325 | 132,40 | 174,40 | **BraRoAZ_10542e3** | 0,088 | 0,0148 | 61,60 | 98,10 |
| **BraRoAZ_10542e3** | 0,030 | 0,1642 | 112,20 | 136,60 | **BraRoAZ_12447e2** | 0,047 | 0,0004 | 101,40 | 54,90 |
